# A diarylheptanoid from *Alpinia officinarum* ameliorates insulin resistance in 3T3-L1 adipocytes and db/db mice through modulation of PPARγ/PI3K/AKT signaling pathway

**DOI:** 10.3389/fphar.2026.1934799

**Published:** 2026-10-14

**Authors:** Qiongshen Chen, Yinghuan Wang, Jie Jiang, Peixia He, Zhenrui Xie, Mingyan Zhou, Zhuoxian Deng, Junqing Zhang, Xuguang Zhang, Qiushi Yin

**Affiliations:** 1 Key Laboratory of Emergency and Trauma of Ministry of Education, Department of Hepatopancreatobiliary Aurgery, The First Affiliated Hospital, Engineering Research Center of Tropical Medicine Innovation and Transformation of Ministry of Education, School of Pharmaceutical Sciences, Hainan Medical University, Haikou, China; 2 Key Laboratory of Tropical Translational Medicine of Ministry of Education, School of Basic Medical Sciences, Hainan Academy of Medical Sciences, Hainan Medical University, Haikou, China; 3 Institute of Clinical Medicine, The Second Affiliated Hospital of Hainan Medical University, Haikou, China

**Keywords:** (R)-5-hydroxy-1,7-diphenyl-3-heptanone, *Alpinia officinarum* hance, insulin resistance, PPARγ/PI3K/AKT, type 2 diabetes mellitus

## Abstract

**Introduction:**

*Alpinia officinarum* Hance and its diarylheptanoid metabolite, (R)-5-hydroxy-1,7-diphenyl-3-heptanone (DPHC), have hypoglycemic and hypolipidemic effects to ameliorate T2DM with insulin resistance (IR). However, the mechanisms by which DPHC ameliorates adipose IR associated with lipid metabolism disorders are not yet fully elucidated.

**Methods:**

DPHC was evaluated in dexamethasone-induced IR-3T3-L1 adipocytes and db/db mice. Exploratory network analysis was used to generate candidate mechanisms.

**Results:**

In IR-3T3-L1 adipocytes, DPHC increased glucose consumption and 2-NBDG uptake, reduced lipid accumulation and inflammatory-marker expression, and increased measured PPARγ and insulin-signaling-associated molecular markers. GW9662 attenuated several cellular effects of DPHC, supporting the involvement of a PPARγ-dependent component. In db/db mice, oral DPHC administration at 80 or 140 mg/kg/day reduced lipid accumulation and improved measured serum lipid-related endpoints. Pocket-directed docking to the DPHC-bound PPARγ ligand-binding domain, together with a single 100-ns molecular-dynamics trajectory, generated a structurally plausible binding pose.

**Conclusion:**

The findings support a testable model in which DPHC may alleviate adipocyte insulin resistance partly through PPARγ-associated PI3K/AKT regulation.

## 1 Introduction

Type 2 diabetes mellitus (T2DM) is a metabolic syndrome caused by disturbances in glucose and lipid metabolism, and it has a major impact on people’s health because it disrupts the balance between energy intake and expenditure (Kalyani et al., 2025; Ding et al., 2025). T2DM is characterized by hyperglycemia and insulin resistance (IR), which is usually associated with chronic complications in multiple organ systems. IR renders the adipose, liver and skeletal muscle tissues less insensitive to endogenous and exogenous insulin, which leads to decreased glucose uptake by the histocytes (Cho et al., 2025; Accili et al., 2025). Moreover, T2DM, as one of the inflammatory diseases, also induces the overproduction of inflammatory cytokines such as tumor necrosis factor-alpha (TNF-α) and interleukin 6 (IL-6) (Moreno-Indias et al., 2014). These cytokines further alter β-cell function through direct action or stimulation of free fatty acid production to affect cellular insulin signaling and lead to T2DM and IR (Oguntibeju, 2019).

Peroxisome proliferator-activated receptor γ (PPARγ) is a ligand-activated nuclear hormone receptor belonging to PPARs family of nuclear hormone receptors. It stimulates adipocytes to secrete adipocytokines such as adiponectin and CCAAT enhancer binding protein α (C/EBPα), enhances insulin sensitivity of peripheral organs and improves chronic inflammation (Shimizu et al., 2006; Katafuchi et al., 2018; He et al., 2003; Chen et al., 2011). Additionally, PPARγ also can activate the phosphatidylinositol 3 kinase (PI3K)/protein kinase B (AKT) signaling pathway to accelerate triglyceride (TG) breakdown in peripheral tissues and inhibit glucagon production, thus improving the disorders of glycolipid metabolism in T2DM (Peng et al., 2015). Furtherly, AKT excitates the expression of downstream glucose transporter 4 (GLUT4) to facilitate glucose transport from outside to inside of cells and enhance insulin sensitivity (Chao et al., 2009; Ramos et al., 2021; Sylow et al., 2021). So the activation of PPARγ/PI3K/AKT signaling pathway can effectively prevent the occurrence and development of IR and T2DM. However, current PPARγ agonists, such as rosiglitazone (Rosi), have severe side effects including edema, weight gain, and cardiovascular risk when taken long-term (Jangra et al., 2025). Therefore, it is necessary to search for new safe and effective activators of PPARγ/PI3K/AKT signaling pathway for the treatment of T2DM.

Numerous plant-derived metabolites have been used as traditional botanical drugs for hundreds of years to treat a variety of diseases such as T2DM (Singh et al., 2025; Liu et al., 2024). The dried rhizome of *Alpinia officinarum* Hance (*A. officinarum*) from the family Zingiberaceae, is a traditional Li medicine in the Hainan province of China, which has been proven to possess hypoglycemic, hypolipidemic, and weight-loss, antioxidant, and other pharmacological activities in modern pharmacological research (Lee et al., 2009). Interestingly, in our previous research, it was found that the diarylheptanoid metabolites in *A. officinarum* showed significant potential in exerting anti-T2DM activity (Li et al., 2022; Zhang, X. G. et al., 2022). DPHC, 5-hydroxy-1,7-diphenyl-3-heptanone, is a diarylheptane metabolite in *A. officinarum*. Our original study revealed that DPHC relieved oxidative stress and adjusted glucose metabolism in high glucose-treated HepG2 cells and db/db mice via activating the Nrf2/ARE-signaling pathway (Zhang X. et al., 2022). Further, it was observed that DPHC treatment increased glucose uptake and ameliorated IR in IR-3T3-L1 adipocytes in our recent pre-experiment. However, the exact mechanism of how DPHC regulates glucolipid metabolism in IR-3T3-L1 cells is currently unknown.

Network analysis can be used as an exploratory approach to generate candidate compound-protein and pathway associations for subsequent experimental evaluation. In the present study, a database-derived network analysis was performed to identify pathways potentially relevant to the effects of DPHC in IR and T2DM. Given the established role of PPARγ-associated PI3K/AKT signaling in adipocyte differentiation, lipid homeostasis, and insulin action, PPARγ-related signaling was selected for further experimental evaluation. We therefore investigated the effects of DPHC on glucose uptake and consumption, lipid accumulation, inflammatory markers, and PPARγ-associated insulin-signaling readouts in dexamethasone-induced insulin-resistant 3T3-L1 adipocytes and db/db mice. The contribution of PPARγ-associated signaling was further assessed using the PPARγ antagonist GW9662, while molecular docking and molecular-dynamics simulations were used to explore a putative structural interaction between DPHC and the PPARγ ligand-binding pocket. This study aimed to provide experimental evidence regarding the metabolic effects of DPHC and to assess whether PPARγ-associated signaling may contribute to these effects.

## 2 Materials and methods

### 2.1 Drug extraction and isolation

DPHC was extracted, isolated and identified from *A. officinarum* Hance by our research group independently, as previously reported (Zhang X. et al., 2022). In brief, *A. officinarum* powders were extracted with ethanol by heating reflux to yield a crude extract. And the crude extract was extracted with ethyl acetate, and then subjected to silica gel CC eluted with petroleum ether/ethyl acetate. The column was eluted in the ratio 15:1 to obtain DPHC, and the tructure was identified as (R)-5-hydroxy-1,7-diphenyl-3-heptanone based on spectroscopic analysis. The purity of DPHC was 98% through HPLC detection. The chemical structure was presented in Figure 1.

**FIGURE 1:**
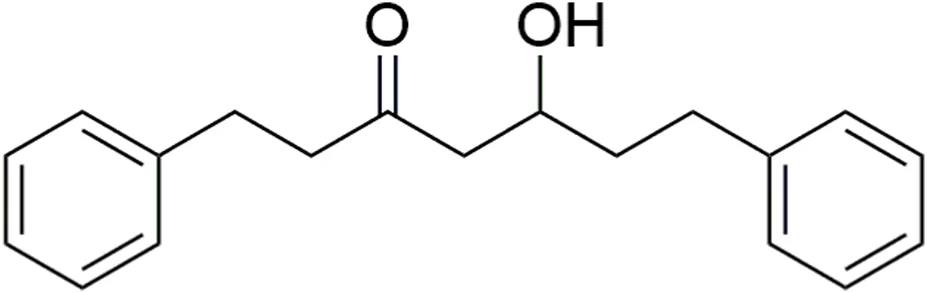
The chemical structure of DPHC.

### 2.2 Materials and reagents

Dulbecco’s modified eagle’s medium (DMEM) and phosphate buffered saline (PBS) were purchased from Gibco-BRL (Grand Island, United States). The newborn calf serum and 3-isobutyl-1-methylxanthine (IBMX) were purchased from Viva Cell (Denzlingen Germany) and Merck KGaA (Sigma, Germany), respectively. The insulin, PPAR antagonist (GW9662), and dexamethasone (Dex) were purchased from selleck (Lanmu, China). The cell counting kit-8 (CCK-8) and 2-[N-(7-nitrobenz-2-oxa-1,3-diazol-4-yl) amino]-2-deoxy-D-glucose (2-NBDG) were obtained from ApexBio (Houston, United States). Glucose determination (glucose oxidase peroxidase method), triglyceride Assay Kit (A110-1-1), and adiponectin Assay Kit (H179-1–2) were obtained from Nanjing Jiancheng Bioengineering Institute (Nanjing, China). Oil Red O Stain Kit (G1262) was obtained from Solarbio (Beijing, China), and Alexa Fluor 488 (A0423) and DAPI (C1002) were obtained from Beyotime Institute of Biotechnology (Haimen, China). Antibodies against Tubulin beta (AF7011), PPAR gamma (AF6284) and Glut 4 (AF5386) were purchased from Affinity (Cambridge, United Kingdom). A 1:100 phosphatase inhibitor dilution and a 1:50 dilution of mixed protease were added to the inhibitor mixture (Abcam, Shanghai, China). Eastep® super total RNA extraction kit was obtained from Shanghai Promage Biological Products Co., Ltd. (Shanghai, China). Hifair III 1st Strand cDNA Synthesis SuperMix and Hieff qPCR SYBR Green Master Mix(No Rox) were purchased from Yeasen Biotechnology (Shanghai, China).

### 2.3 Exploratory network analysis

#### 2.3.1 Candidate targets of DPHC and insulin resistance in T2DM

Putative human targets of DPHC were retrieved in the original analysis from SwissTargetPrediction (probability >0) and PharmMapper using the two-dimensional DPHC structure. Disease-associated genes were retrieved with the terms “type 2 diabetes” and “insulin resistance” from PharmGKB, GeneCards, TTD, and OMIM; duplicate identifiers were removed after mapping to standardized human gene symbols. The archived materials available for this revision did not retain complete query dates, database versions, thresholds for every source, or unfiltered exports. The resulting lists were therefore treated as historical, exploratory outputs and not as an independently reproducible validation set. Adding further correlated prediction databases was not considered a substitute for experimental target-engagement evidence.

#### 2.3.2 Protein-protein interaction graphs

Overlapping candidate genes were submitted to STRING for a descriptive protein–protein interaction network and imported into Cytoscape 3.8.2. CytoNCA centrality measures (degree, betweenness, and closeness) were used to summarize network topology. Centrality was not interpreted as target accuracy, causal importance, or evidence of compound–protein binding.

#### 2.3.3 GO enrichment and KEGG pathway analysis

GO and KEGG enrichment of the overlapping candidate genes was performed with Metascape, using P < 0.05 in the original analysis. The top 10 GO terms within biological process, molecular function, and cellular component categories and 20 representative KEGG pathways were visualized. Because the background gene universe and multiple-testing metadata were not retained in the archived files, enrichment results were interpreted descriptively rather than confirmatorily.

#### 2.3.4 Building component targets and pathways in a network diagram

Cytoscape 3.8.2 was used to visualize a component–target–pathway network linking DPHC, overlapping candidate genes, and representative KEGG pathways. Node degree was reported as a topological descriptor only. PPARγ-associated signaling was carried forward as a biological hypothesis and was evaluated experimentally; the network itself was not considered validation.

### 2.4 Molecular docking

Docking was used to generate candidate poses, not to estimate binding affinity. The human PPARγ ligand-binding domain co-crystal structure with rosiglitazone (PDB 7AWC; chain A; 1.74 Å resolution) was used. Water molecules and the co-crystallized ligand were removed, polar hydrogens and charges were added, and receptor and ligands were converted to PDBQT format. DPHC, rosiglitazone (positive control), and the close analogue 5-methoxy-1,7-diphenyl-3-heptanone were docked with AutoDock Vina 1.2.2 (Trott and Olson, 2010). The search box was centered at x = 41.46, y = 4.65, and z = 83.38 Å with dimensions 15 Å × 15 Å × 15 Å, corresponding to the crystallographic ligand-binding pocket. Nine output modes were retained. The random seeds recorded in the supplied logs were 1720507008 for DPHC, 1751794928 for rosiglitazone, and −1620842248 for the analogue. Vina scores were interpreted only as scoring-function outputs; no score was treated as a binding free energy, dissociation constant, or proof of specificity.

### 2.5 Molecular dynamics

A single 100-ns all-atom molecular-dynamics simulation was performed for the selected DPHC-PPARγ pose using GROMACS 2022.2 (Abraham et al., 2015). The protein used the Amber99SB-ILDN force field (Lindorff-Larsen et al., 2010); ligand parameters were generated with GAFF2 (Wang et al., 2004) using the supplied RESP-derived charges. The complex was solvated with TIP3P water in a periodic box with a 1.0-nm solute-to-box distance and 0.15 M NaCl. Energy minimization comprised 3,000 steepest-descent and 2,000 conjugate-gradient steps. Production was run in the NPT ensemble at 310 K and 1 bar with a 2-fs time step, particle-mesh Ewald electrostatics (1.0-nm real-space cutoff), LINCS bond constraints, Nosé–Hoover temperature coupling (0.1 ps), and Parrinello–Rahman pressure coupling (2 ps). Backbone RMSD, Cα RMSF, radius of gyration, solvent-accessible surface area, protein–ligand hydrogen bonds, and a two-dimensional RMSD/radius-of-gyration free-energy landscape were calculated with GROMACS tools. The supplied archive did not contain replicate trajectories or an independently reproducible binding-free-energy calculation; none is claimed.

### 2.6 Cell culture and treatment

The murine fibroblast cell line 3T3-L1 was obtained from Shanghai Biotechnology Co. Ltd. and cultured under optimal condition of 37 °C with 5% CO_2_ in the cell culture incubator. The DMEM culture medium utilized for the maintenance of the cells was supplemented with 10% calf serum and 1% penicillin-streptomycin. In order to facilitate the process of cell fusion, 3T3-L1 cells were plated into multiwell plates and allowed to incubate for a period of 3 days. After the initial incubation, the culture medium was replaced with DMEM containing 10% FBS alongside 10 μg/mL insulin. The cells continued to be cultivated in this enriched DMEM for an additional 4 days, during which the medium also included 0.5 mM IBMX and 1 μM Dex. This carefully orchestrated regimen was instrumental in guiding the cells towards differentiation. Ultimately, the cells were maintained in DMEM supplemented with 10% FBS until approximately 90% of them displayed defining characteristics associated with the adipocyte phenotype, indicating successful differentiation.

Then, the IR model was created by exposing 3T3-L1 cells to 1 µM Dex for 96 h (Yuan et al., 2019) and subsequently treating them with 30, 50, and 70 µM DPHC or 20 µM positive control rosiglitazone (Rosi) for 16 h to investigate the impact of DPHC on IR. Following treatment, the cells were classified into several groups: CON group (DMEM), MOD group (DMEM +1 µM Dex), Rosi group (DMEM +1 µM Dex +20 µM Rosi), and DPHC treatment groups (DMEM +1 µM Dex +30, 50, 70 µM DPHC). Moreover, a PPARγ antagonist (GW9662, 30 μM) constituted an additional experimental group both with and without DPHC (30 µM). Finally, cells were harvested for further analysis.

### 2.7 Analysis of cell viability

Cell viability was assessed via the Cell Counting Kit-8. In this experiment, 3T3-L1 preadipocytes were cultured in 96-well plates at a specified density of 1 × 10^4^ cells per well, supplemented with 10% calf serum in a DMEM environment. After allowing for a 24 h incubation period, the adhering cells were treated with different concentrations of DPHC, which varied between 1 and 80 μM. Following this treatment, 10 μL of the CCK-8 solution was added to each well, and the plates were incubated again at a temperature of 37 °C for additional 1 h. The absorbance from each well was measured at a wavelength of 450 nm using the SpectraMax Plus automated enzyme labeling instrument (Molecular Devices, Sunnyvale, CA, United States).

### 2.8 Analysis of glucose consumption and uptake

Glucose consumption was tested using a glucose detection kit. Successfully differentiated 3T3-L1 adipocytes were inoculated into 96-well plates and cultured in an incubator. When the single-cell layer reached 80%–90% confluence, the cells were administered according to the above groups. After 24 h, 2 μL of the cell culture solution was aspirated from the 96-well plate, mixed well with 200 μL of working solution, and incubated for 10 min. A SpectraMax Plus automated enzyme labeling instrument was used to measure the absorbance at 500 nm. Cellular glucose consumption was expressed as: Glucose consumption = Glucose concentration in blank group (no cells, only medium) - Glucose concentration in each group. Meanwhile, the number of cells was corrected by adding CCK-8 solution to each well for 1 h to measure OD value.

2-NBDG, a fluorescent analog of glucose, was utilized to assess glucose uptake by cells. The procedure employed for this assessment was adapted from the methodology to fit the specific experimental need of this study (Paprocki et al., 2025). In brief, cells were cultured at a density of 2 × 10^5^ cells/mL in 6-well plates. After the preparation and administration of the treatment, 2-NBDG was introduced to the cells and allowed to incubate at 37 °C for a period of 20 min, achieving a final concentration of 25 μM. Subsequently, the cells were suspended in PBS and underwent two washes to remove any unbound fluorescent dye. The fluorescence intensity was then measured utilizing a flow cytometer, specifically the FACS Calibur from Agilent NovoCyte Penteon, United States. Ultimately, the quantification of fluorescence provided an effective estimate of the total cellular glucose uptake.

### 2.9 Oil Red O staining

The Oil Red O staining method was employed to determine the effect of DPHC on lipid droplet size and adipogenesis in 3T3-L1 adipocytes, adipose and liver tissues of db/db mice. For this procedure, 12-well plates were used to cultivate 3T3-L1 preadipocytes until they differentiated. Following IR induction and treatment with Rosi or DPHC, differentiated 3T3-L1 adipocytes were fixed for 15 min with 10% formaldehyde. Mouse adipose and liver tissues were fixed, embedded, frozen sectioned and then fixed in 4% paraformaldehyde. The cells and tissues were subsequently incubated for 30 min with filtered oil Red O working solution (Oil Red O staining A: Oil Red O staining B = 3:2), washed with 60% isopropanol until they became colorless, and examined using Zeiss’s AxioVertA 1 inverted microscope (Oberkochen, Germany). Micrographs were acquired to document the research results, and the quantification of lipid droplet was carried out using the ImageJ software.

### 2.10 Determination of adiponectin and TG levels

After induction of IR and pretreatment of the DPHC and Rosi, 3T3-L1 adipocyte suspension was diluted with PBS to achieve a cell concentration of 10^6^ cells/mL. The cells were damaged and the contents within them were released by repeatedly freezing and thawing. Collecting the cell supernatant with a sterile tube after centrifuging 3,000 rpm for 20 min, and the adiponectin level in the cell supernatant of all groups was determined using a Adiponectin Assay Kit in 3T3-L1 adipocytes.

3T3-L1 adipocytes were harvested using the ice-cold PBS to measure intracellular TG level. In a brief, the cells were lysed, and then 1 mL of the TG extraction reagent consisting n-heptane and isopropanol was introduced to the lysate. After the addition of the extraction reagent, the lysate underwent centrifugation at 4 °C to separate the supernatant, which contained the extracted TG. Finally, the TG concentration in the supernatant was accurately measured using a TG assay kit.

### 2.11 Immunofluorescence assay

3T3-L1 preadipocytes were cultured in a confocal dish until differentiation. After the induction of IR and pretreatment with DPHC or Rosi, the cells were washed with ice-cold PBS and fixed with 4.0% paraformaldehyde for 15 min. After washing with PBS, the cells were treated with 0.2% Triton X-100 for 20 min, followed by five washes with PBS. Then, the cells were blocked with goat serum for 30 min at 25 °C and incubated with a rabbit polyclonal antibody against PPARγ (1:300 dilutions in Immunol Staining Primary Antibody Dilution Buffer) at 4 °C overnight. After five washes with PBS, the cells were incubated with Anti-Rabbit Alexa Fluor 488 (1:300 dilutions in QuickBlock™ Secondary Antibody Dilution Buffer) at 25 °C for 1 h. Following a wash with PBS, the cells were stained with DAPI for 15 min at 25 °C and removed excess dye with PBS wash. Images were captured using an Olympus confocal microscope (FV3000; Olympus Corporation, Japan).

### 2.12 Db/db mice experimentation

The db/db mice experimentation was conducted based on our previous research and modified accordingly (Zhang X. et al., 2022). Briefly, 7-week-old male C57BL/KsJ db/db and db/m mice provided by Jiangsu Jicui Yaokang Biotechnology Co., Ltd in China (Animal quality certificate No. B202511280312), were given unrestricted access to both food and water at the Experimental Animal Center of Hainan Medical University. The mice were kept in a stable temperature of 25 °C and 12 h light-dark cycle to simulate natural conditions. All experimental procedures were implemented under the approval of the Experimental Animal Ethics Committee of Hainan Medical University (reg. no. HYLL-2021-377), and carried out in accordance with the Animal Ethics Regulations of the People’s Republic of China and the Animal Ethics Guidelines of the People’s Republic of China. Following a week of acclimatization, nine male db/m mice and db/db mice were randomly respectively distributed to the blank control group and the db/db model group, while the remaining 27 db/db mice were randomly assigned to the other three experimental groups. These db/db mice initially served as a model organism and were kept without any treatment during the preliminary phase. In the positive control group, db/db mice received treatment with Rosi at a dosage of 10 mg/kg of body weight. Based on the preliminary efficacy range of 70–160 mg/kg and the findings of our previous 8-week study in db/db mice (Zhang X. et al., 2022), DPHC was administered once daily to db/db mice at doses of 80 or 140 mg/kg/day. These doses were selected for preclinical efficacy evaluation and were not intended to establish a clinical dose. All mice received oral gavage at a volume of 0.2 mL/10 g body weight. The drugs were dissolved in 1% sodium carboxymethyl cellulose solution, while mice in the db/m and db/db groups received an equivalent volume of vehicle. The animal feed was provided by the Guangdong Medical Laboratory Animal Center. In addition, dosing was administered once daily at a fixed time for eight consecutive weeks. After the experimental period, adipose tissue samples were collected from the mice post-mortem using the cervical dislocation method to carry out the subsequent analysis.

### 2.13 RNA extraction and qPCR analysis

QPCR was used to quantify mRNA expression in differentiated 3T3-L1 adipocytes and adipose tissues collected from db/db mice. Total RNA was extracted using the Eastep® Super Total RNA Extraction Kit according to the manufacturer’s instructions and reverse-transcribed into cDNA using Hifair® III 1st Strand cDNA Synthesis SuperMix. QPCR was performed using Hieff® qPCR SYBR Green Master Mix (No ROX) on a CFX96 Touch Real-Time PCR Detection System (Bio-Rad). The amplification program comprised an initial denaturation at 95 °C for 5 min, followed by 40 cycles of denaturation at 95 °C for 10 s and annealing/extension at 60 °C for 30 s. A melting-curve analysis was performed after amplification to verify product specificity. β-actin was used as the reference gene. For relative quantification, the CON group in the cell experiments and the db/m group in the animal experiments were used as the respective calibrators. Relative mRNA expression was calculated using the 2^−ΔΔCT^ method.

### 2.14 Western blot analysis

The RIPA buffer was used to extract all soluble proteins from 3T3-L1 adipocytes and adipose tissue of db/db mice. A 1:100 phosphatase inhibitor dilution and a 1:50 dilution of mixed protease were added to the inhibitor mixture. After mixing the lysate (20 µg protein) with 5 × SDS-PAGE sample buffer, it was boiled for 10 min at 100 °C. The protein bands in the 10% gel were then electrotransferred onto a polyvinylidene difluoride membrane after the samples had been separated using SDS-PAGE. Tris-buffered saline containing 0.5% (v/v) Tween 20 (TBST) was used thrice after the membranes were sealed with 5% skim milk in Tris buffer for 1 h at room temperature. The blot was then washed three more times with TBST after incubating overnight at 4 °C with the appropriate primary antibody. The blot was subsequently washed thrice with TBST after incubating for 1 h at room temperature with the proper secondary antibody. Finally, it was examined using Image Lab analysis equipment (Bio-Rad, ChemiDocXRS+, United States) after identifying positive bands with the ECL plus western blot detection reagent. The microtubule protein levels were used to determine the protein expressions.

### 2.15 Immunohistochemical analysis

The paraffin sections of adipose and liver tissues of db/db mice were deparaffinized with xylene, hydrated with gradient ethanol, soaked with PBS buffer, and placed in EDTA antigen recovery solution. The sections were drip-added with 3% H_2_O_2_ solution, incubated in a dark place at room temperature for 15 min, and rinsed with PBS. The adipose tissue sections were incubated with PPARγ antibody, and the liver tissue sections were incubated with IRS1 antibody for primary antibody overnight, and the secondary antibody was incubated at room temperature for 30 min. Freshly prepared DAB color solution was added drop by drop, and when the positive signal was brown and yellow, the color was immediately immersed in distilled water to stop the color development. The nuclei were counterstained with hematoxylin for 1 min and rinsed with running water to return to blue. After dehydration with gradient ethanol and transparency with xylene, slices were sealed with neutral gum and allowed to dry in a fume hood. The results were obtained by ImageJ software analysis.

### 2.16 Statistical analysis

All values were expressed as mean ± standard deviation (SD). Statistical analyses among the groups were performed using one-way ANOVA followed by GraphPad Prism 10.1.2 software. P < 0.05 was considered statistically significant.

## 3 Results

### 3.1 Exploratory network analysis

#### 3.1.1 Database-derived candidate targets of DPHC and insulin resistance

The historical SwissTargetPrediction and PharmMapper outputs contained 369 unique DPHC-associated candidate genes after deduplication. Disease-database outputs were combined to obtain 1,063 unique genes associated with both T2DM and insulin resistance, of which 79 overlapped the DPHC-associated list (Figures 2A–D). These counts summarize database retrieval and overlap; they do not estimate sensitivity, specificity, binding, or therapeutic relevance.

**FIGURE 2:**
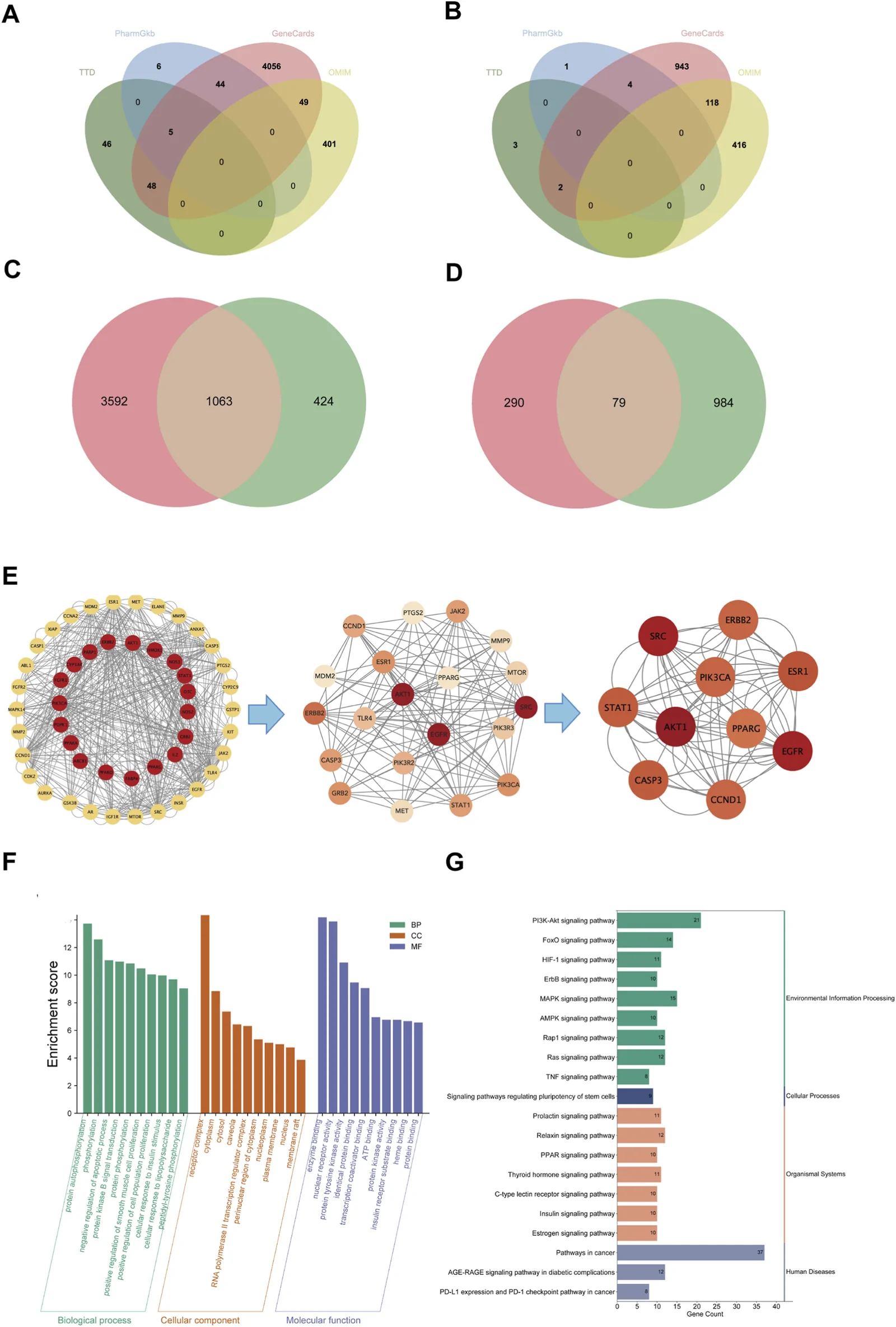
Exploratory network analysis of DPHC, insulin resistance, and T2DM. **(A–D)** Database-retrieval and overlap summaries. **(E)** STRING-derived PPI network and high-centrality nodes. **(F)** Descriptive GO enrichment. **(G)** Representative KEGG annotations. The analysis was hypothesis-generating; overlap, enrichment, and centrality do not establish target accuracy, binding, or causality.

#### 3.1.2 Protein–protein interaction (PPI)

The 79 overlapping genes formed a STRING-derived network of 79 nodes and 841 edges after removal of unconnected nodes (Figure 2E). AKT1, SRC, EGFR, PIK3CA, and PPARG had degree values of 33, 30, 30, 22, and 20, respectively. These hubs are highly studied signaling proteins, and their centrality may reflect literature and database structure. They were therefore treated as candidates rather than “core therapeutic targets.”

#### 3.1.3 Analysis of GO enrichment and KEGG pathways

Exploratory GO analysis of the 79 overlapping genes returned 598 terms (458 biological process, 49 cellular component, and 91 molecular function terms; Figure 2F). Representative annotations included protein phosphorylation, AKT signaling, insulin responses, receptor complexes, and kinase-related functions. These enrichments describe annotation patterns among the submitted genes and do not establish that DPHC regulates the terms or pathways.

KEGG annotation returned 138 pathways; 20 representative pathways were visualized (Figure 2G), including PI3K/AKT, MAPK, PPAR, and TNF signaling. PPARγ-associated insulin signaling was selected for experimental study because it was biologically relevant to adipocyte insulin resistance and could be challenged pharmacologically with GW9662. This selection should not be interpreted as confirmation of the database ranking.

### 3.2 *In vitro* assays

#### 3.2.1 Effect of DPHC on the viability of 3T3-L1 preadipocytes

The cell viability was estimated through CCK-8 assay to determine the toxicity and dosing concentration of DPHC on 3T3-L1 preadipocytes (Figure 3A). The result indicated that DPHC did not exert a significant effect on the viability of 3T3-L1 preadipocytes at concentrations of 1–80 μM for 16 h. Therefore, we selected DPHC at doses of 30, 50, and 70 μM for subsequent experiments.

**FIGURE 3:**
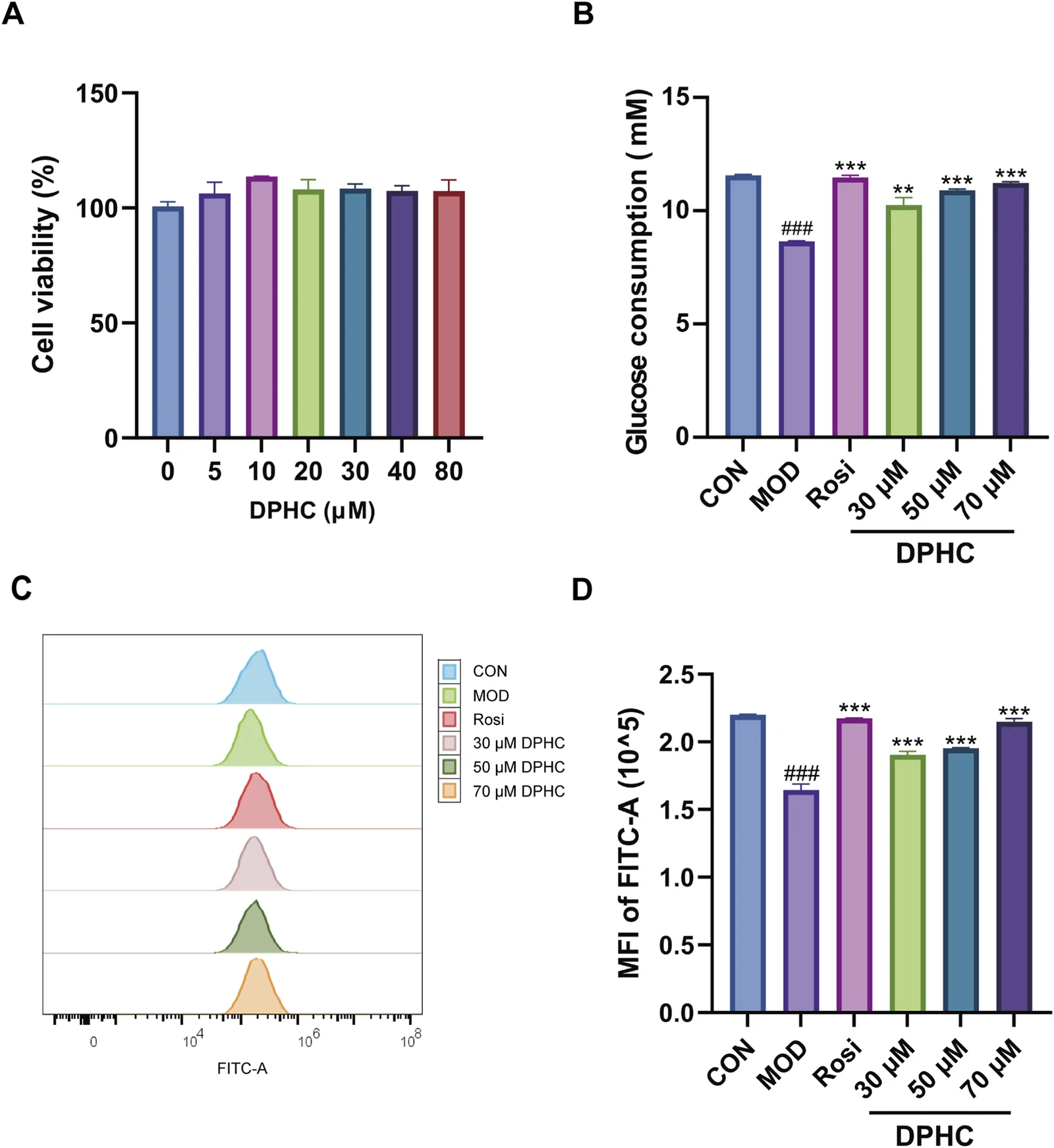
Effects of DPHC on cell viability, glucose consumption and uptake in IR-3T3-L1 adipocytes. **(A)** Cell viability after treatment with different concentrations of DPHC. **(B)** The effect of DPHC on glucose consumption. **(C)** Flow cytometry histograms evaluating glucose uptake (measured by FITC-A fluorescence). **(D)** Quantitative analysis of the mean fluorescence intensity (MFI) for glucose uptake. All data are expressed as the mean ± SD (n ≥ 3). ^###^P < 0.001 vs. the CON group; ^***^P < 0.001 and ^**^P < 0.01 vs. the MOD group.

#### 3.2.2 Effect of DPHC on glucose consumption and uptake in IR-3T3-L1 adipocytes

The IR-3T3-L1 cells were induced using 1 μM Dex, and the improvement effect of DPHC on IR in IR-3T3-L1 adipocytes was analyzed by measuring glucose consumption and uptake. After 96 h of induction with 1 μM Dex, glucose consumption in 3T3-L1 adipocytes was significantly decreased (P < 0.001). Moreover, glucose uptake in the MOD group was considerably lower than that in the CON group, indicating that the IR cell model had been successfully established (Figures 3B–D). However, compared with the MOD group, treatment with DPHC led to a dose-dependent increase in glucose consumption, similar to the effect observed with Rosi (P < 0.001) (Figure 3B). Similarly, treatment with DPHC at concentrations ranging from 30 to 70 μM reversed the decrease in glucose uptake in a dose-dependent manner (P < 0.001). Notably, a dose of 70 μM DPHC restored glucose uptake to a level nearly comparable to normal (P < 0.001) (Figures 3C,D). These observations indicated that DPHC enhanced both glucose consumption and uptake in IR-3T3-L1 adipocytes; therefore, DPHC contributed to the improvement of insulin sensitivity and glucose metabolism.

#### 3.2.3 Effect of DPHC on lipid accumulation in IR-3T3-L1 adipocytes

The role of DPHC on lipid accumulation was investigated in IR-3T3-L1 adipocytes. The Oil Red O staining showed that differentiated 3T3-L1 adipocytes in the CON group contained markedly more lipid droplets than the undifferentiated cells, confirming successful adipocyte differentiation (Figure 4A). In contrast, the MOD group, which exhibited IR, presented with larger and more numerous lipid droplets compared to the CON group. This observation suggested that IR exacerbated lipid accumulation in 3T3-L1 adipocytes. As expected, treatment with the positive control Rosi markedly reduced both the size and number of lipid droplets relative to the MOD group. Similarly, treatment with DPHC at 30–70 μM reduced lipid-droplet accumulation, with noticeably smaller and fewer lipid droplets observed than in the MOD group (Figure 4A). Consistent with the staining results, the quantified lipid level was significantly increased in the MOD group and was reduced by Rosi and DPHC treatment (Figure 4B). Besides, the MOD group showed significantly elevated triglyceride (TG) content in comparison to the CON group (Figure 4C), reinforcing the conclusion that IR was a contributory factor in lipid accumulation in 3T3-L1 adipocytes. Importantly, after treatment with 30–70 μM of DPHC, TG contents in IR-3T3-L1 adipocytes significantly decreased (P < 0.01), which corroborated the observations made in the Oil Red O staining. Collectively, these findings highlighted the capacity of DPHC to effectively diminish lipid accumulation in IR-3T3-L1 adipocytes.

**FIGURE 4:**
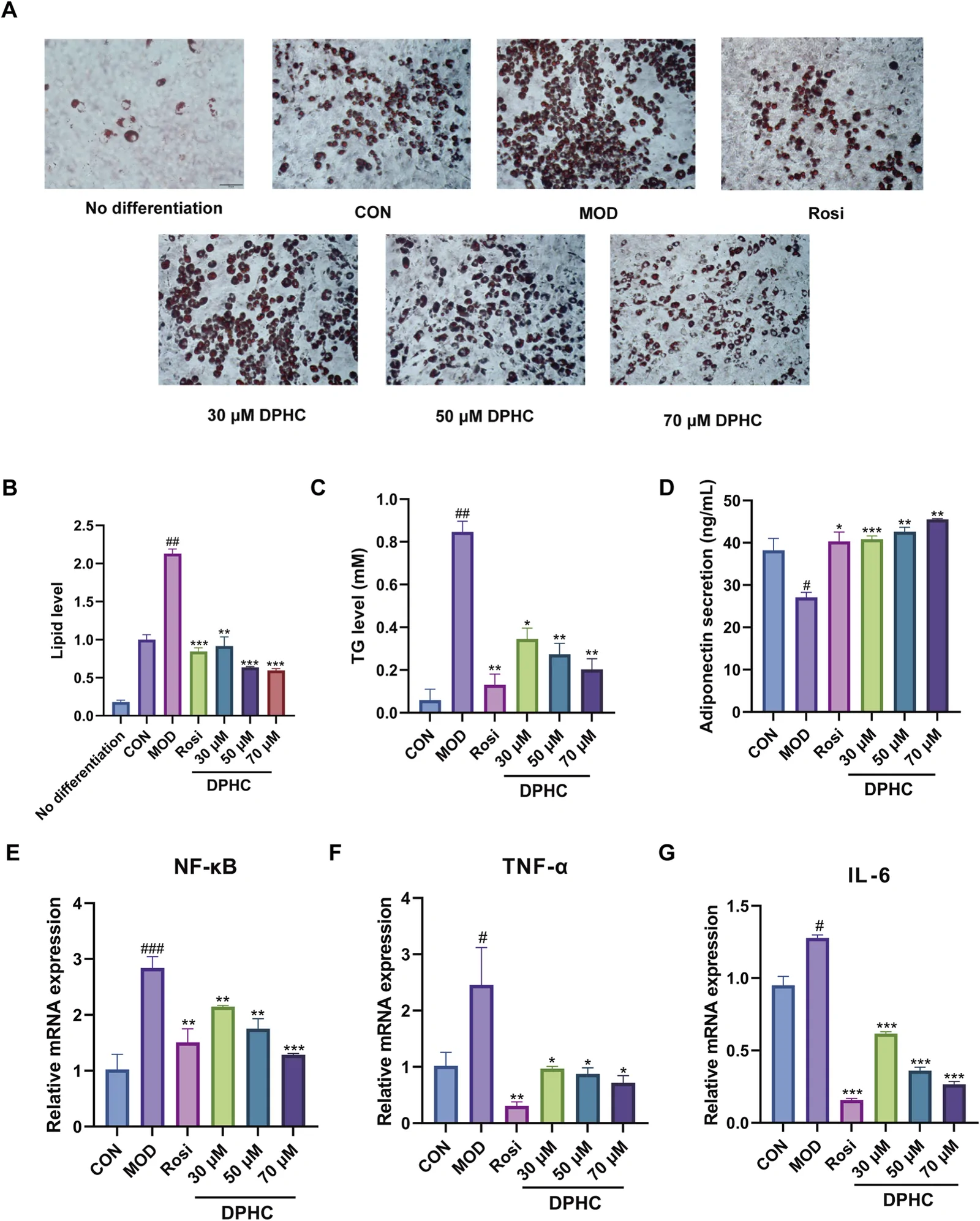
Effects of DPHC on lipid accumulation, adiponectin secretion, and inflammatory factor expressions in IR-3T3-L1 adipocytes. **(A)** Lipid accumulation was visualized by Oil Red O staining (magnification: ×100). **(B)** Quantification of the lipid-droplet accumulation for Oil Red O staining. **(C)** TG level. **(D)** Adiponectin secretion level. **(E–G)** qPCR analysis. Relative mRNA expressions of NF-κB **(E)**, TNF-α **(F)**, and IL-6 **(G)**. All data are expressed as the mean ± SD (n = 3). ^###^P < 0.001, ^##^P < 0.01 and ^#^P < 0.05 vs. the CON group; ^***^P < 0.001, ^**^P < 0.01 and ^*^P < 0.05 vs. the MOD group.

#### 3.2.4 Effect of DPHC on the adipokines in IR-3T3-L1 adipocytes

The regulation of glucose and lipid balance involved the release of various cytokines from adipocytes. When the secretion function of adipocytes changed, it led to an increase in the production of cytokine and pro-inflammatory substances, such as NF-κB, TNF-α and IL-6 (Karampetsou et al., 2022; Wang et al., 2022). And elevated TG level also contributed to the heightened release of inflammatory mediators. And these adipokines played a crucial role in maintaining glycolipid metabolism. In this study, the adiponectin secretion was measured by a enzyme-linked immunosorbent assay and intracellular mRNA levels of important markers involved in inflammatory response and metabolic regulation, specifically NF-κB, TNF-α and IL-6 were evaluated using qPCR method. The results demonstrated a notable decline in the adiponectin secretion (P < 0.05) within MOD group when compared to the CON group, indicating a compromised lipid metabolism under IR conditions (Figure 4D). In contrast, DPHC treatment led to a substantial increase in the secretion of intracellular adiponectin (P < 0.01), a protein involved in lipid transport and metabolism. Moreover, DPHC significantly reduced pro-inflammatory cytokines NF-κB, TNF-α and IL-6 mRNA levels (P < 0.05), further highlighting its potential in mitigating inflammatory responses associated with IR (Figures 4E–G). Hence, DPHC had a beneficial effect on glucolipid metabolism by enhancing adiponectin secretion and modulating inflammatory cytokines.

#### 3.2.5 Effect of DPHC on the PPARγ/PI3K/AKT signaling pathway in IR-3T3-L1 adipocytes

Guided by the exploratory analysis, we measured PPARγ and selected insulin-signaling-associated molecules in IR-3T3-L1 adipocytes. DPHC increased PPARγ immunofluorescence and increased total PPARγ, PI3K, AKT, and GLUT4 protein abundance relative to the model group (Figures 5A–C). It also increased PPARγ, C/EBPα, IRS1, PI3K, AKT, and GLUT4 mRNA abundance (Figures 5D–I). These abundance measurements are consistent with increased signaling capacity but do not directly measure PPARγ transcriptional activity, PI3K activity, AKT phosphorylation, or GLUT4 translocation.

**FIGURE 5:**
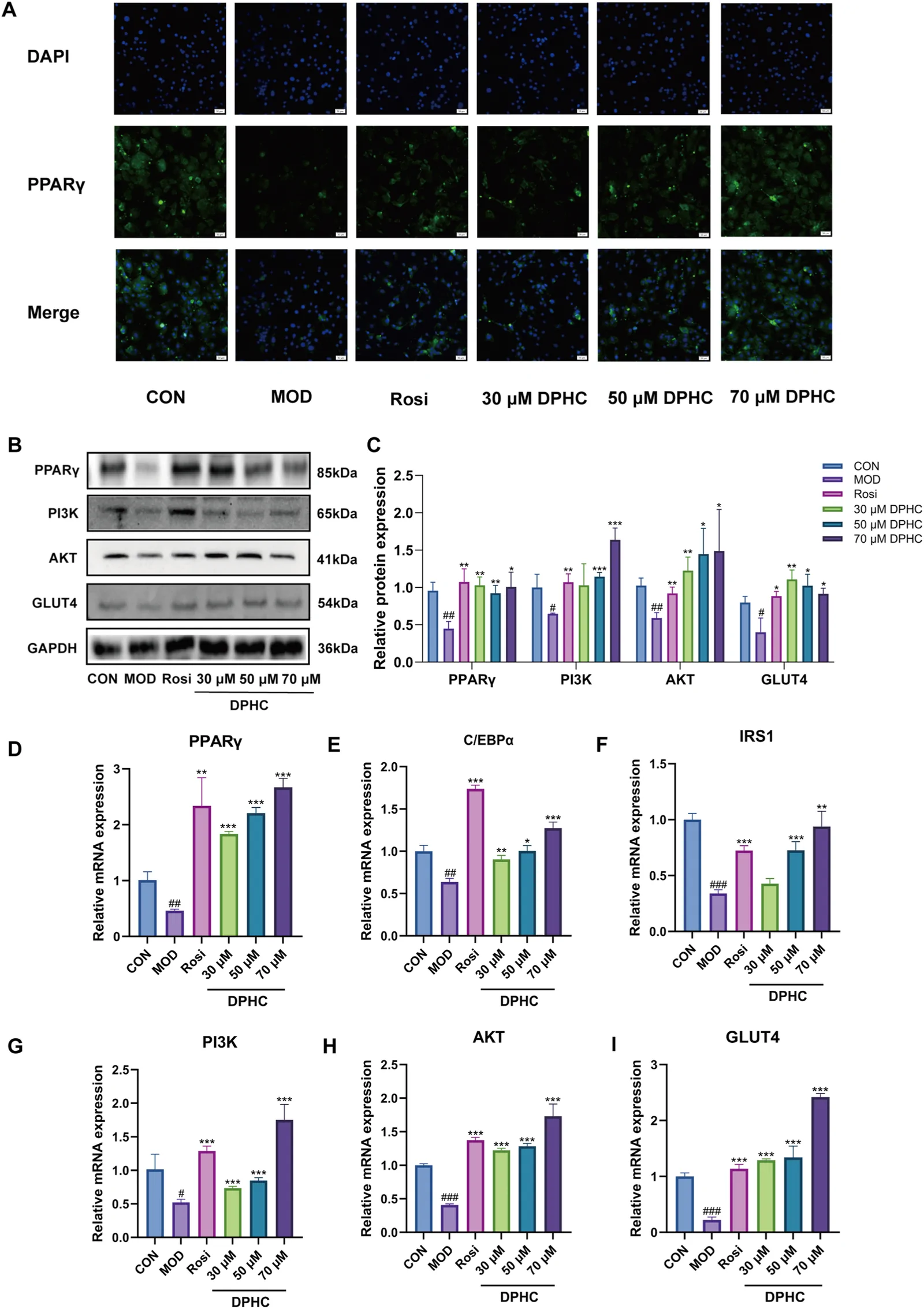
Effect of DPHC on the PPARγ/PI3K/AKT signaling pathway in IR-3T3-L1 adipocytes. **(A)** Immunofluorescence staining of PPARγ (scale bar = 50 μm). **(B)** Western blot analysis. PPARγ, PI3K, AKT, and GLUT4 protein expressions. **(C)** Quantitative analysis of PPARγ, PI3K, AKT, and GLUT4 protein expressions. **(D–I)** qPCR analysis. Relative mRNA expressions of PPARγ, C/EBPα, IRS1, PI3K, AKT, and GLUT4. All data are expressed as the mean ± SD (n = 3). ^###^P < 0.001, ^##^P < 0.01 and ^#^P < 0.05 vs. the CON group; ^***^P < 0.001, ^**^P < 0.01 and ^*^P < 0.05 vs. the MOD group.

#### 3.2.6 Pocket-directed docking generated a candidate PPARγ pose, and PPARγ inhibition blunted cellular effects of DPHC

Pocket-directed docking to the rosiglitazone-bound PPARγ ligand-binding domain yielded best-mode Vina scores of −7.8 kcal/mol for DPHC, −8.5 kcal/mol for rosiglitazone, and −7.8 kcal/mol for 5-methoxy-1,7-diphenyl-3-heptanone (Figure 6A). The equal score for DPHC and the close analogue indicates that this calculation did not discriminate the compounds and cannot establish specificity. The modeled DPHC pose occupied the canonical ligand-binding pocket and provided a structural hypothesis for further testing. A single 100-ns molecular-dynamics trajectory of this pose showed a post-10-ns mean backbone RMSD of 0.269 ± 0.015 nm and an overall mean radius of gyration of 1.941 ± 0.008 nm. Mean solvent-accessible surface area was 145.8 ± 2.6 nm^2^ and the mean protein–ligand hydrogen-bond count was 0.71 ± 0.47. The free-energy landscape contained a dominant basin with adjacent conformational states (Supplementary Figure S1). These results indicate persistence of the starting pose within one simulation; they do not measure binding affinity or target engagement.

**FIGURE 6:**
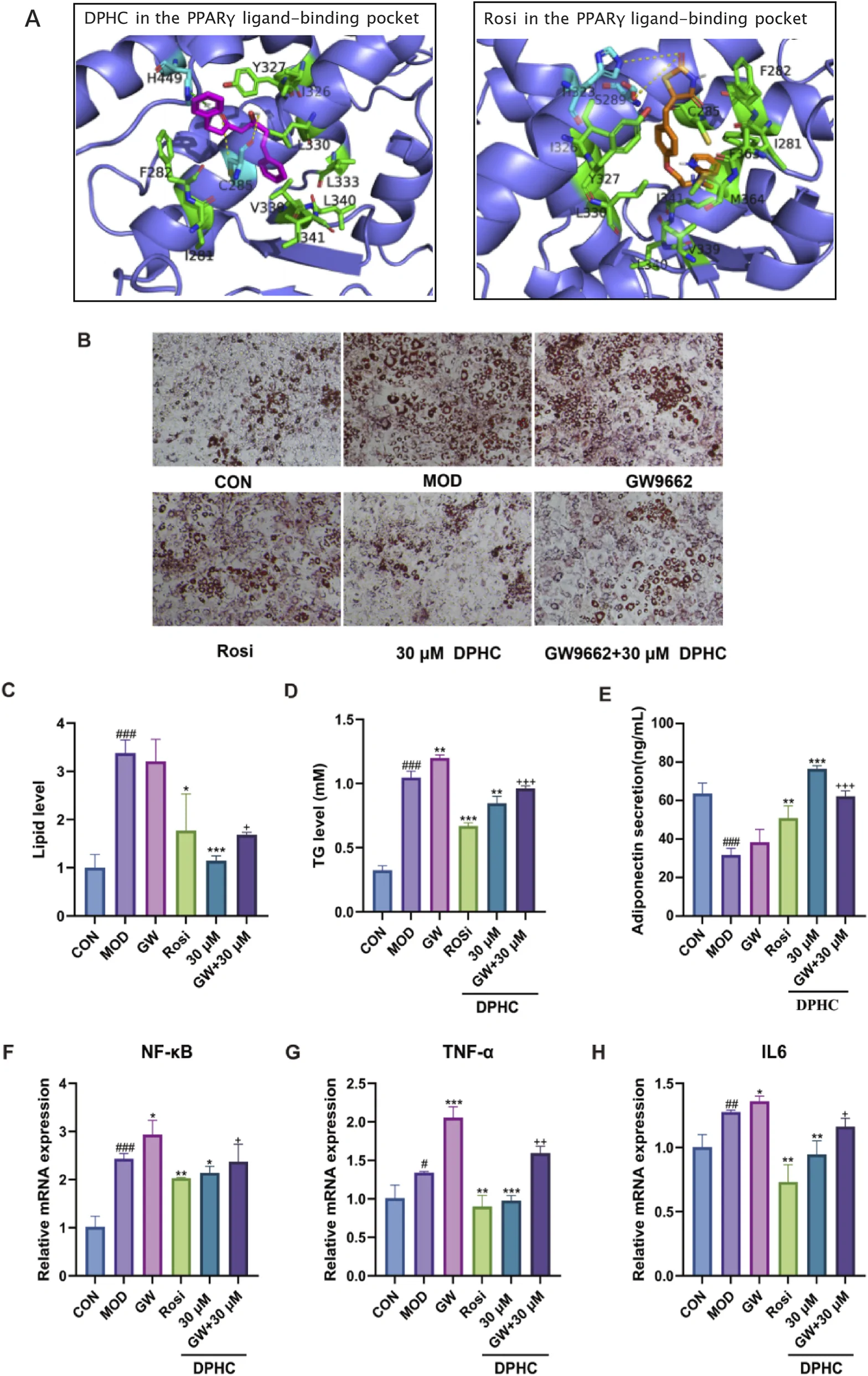
Pocket-directed docking and pharmacologic PPARγ inhibition. **(A)** Candidate poses of DPHC and rosiglitazone in the crystallographic PPARγ ligand-binding pocket (PDB 7AWC); labels report AutoDock Vina scores, not binding free energies or affinities. The close analogue 5-methoxy-1,7-diphenyl-3-heptanone had the same best score as DPHC (−7.8 kcal/mol; not shown). **(B)** Lipid accumulation was visualized by Oil Red O staining (magnification: ×100). **(C)** Quantification of the lipid-droplet accumulation. **(D)** TG level. **(E)** Adiponectin secretion level. **(F–H)** qPCR analysis. Relative mRNA expressions of NF-κB **(F)**, TNF-α **(G)**, and IL-6 **(H)**. All data are expressed as the mean ± SD (n = 3). ^###^P < 0.001, ^##^P < 0.01 and ^#^P < 0.05 vs. the CON group; ^***^P < 0.001, ^**^P < 0.01 and ^*^P < 0.05 vs. the MOD group; ^+++^P < 0.001, ^++^P < 0.01 and ^+^P < 0.05 vs. the 30 μM DPHC group.

GW9662 was used to test whether selected cellular effects of DPHC required PPARγ function. GW9662 alone did not significantly alter the measured lipid endpoints relative to the model group. DPHC reduced Oil Red O staining and TG content, whereas co-treatment with GW9662 blunted these effects (Figures 6B–D). DPHC also increased adiponectin and decreased NF-κB, TNF-α, and IL-6 mRNA; these changes were attenuated by GW9662 (Figures 6E–H). The pharmacologic interaction supports a PPARγ-dependent component but, because GW9662 may have context-dependent effects and no direct binding assay was performed, it does not demonstrate that DPHC binds PPARγ.

#### 3.2.7 DPHC modulated insulin-signaling-associated expression through a PPARγ-dependent mechanism in IR-3T3-L1 adipocytes

In IR-3T3-L1 adipocytes, DPHC increased PPARγ immunofluorescence and the measured protein and mRNA abundance of PPARγ-associated insulin-signaling components; co-treatment with GW9662 attenuated these changes (Figures 7A–I). The results support functional dependence on PPARγ under the tested conditions. Because only total PI3K and AKT abundance was measured, the data do not establish kinase activation; because no ligand-binding or target-engagement assay was performed, they do not establish direct interaction between DPHC and PPARγ.

**FIGURE 7:**
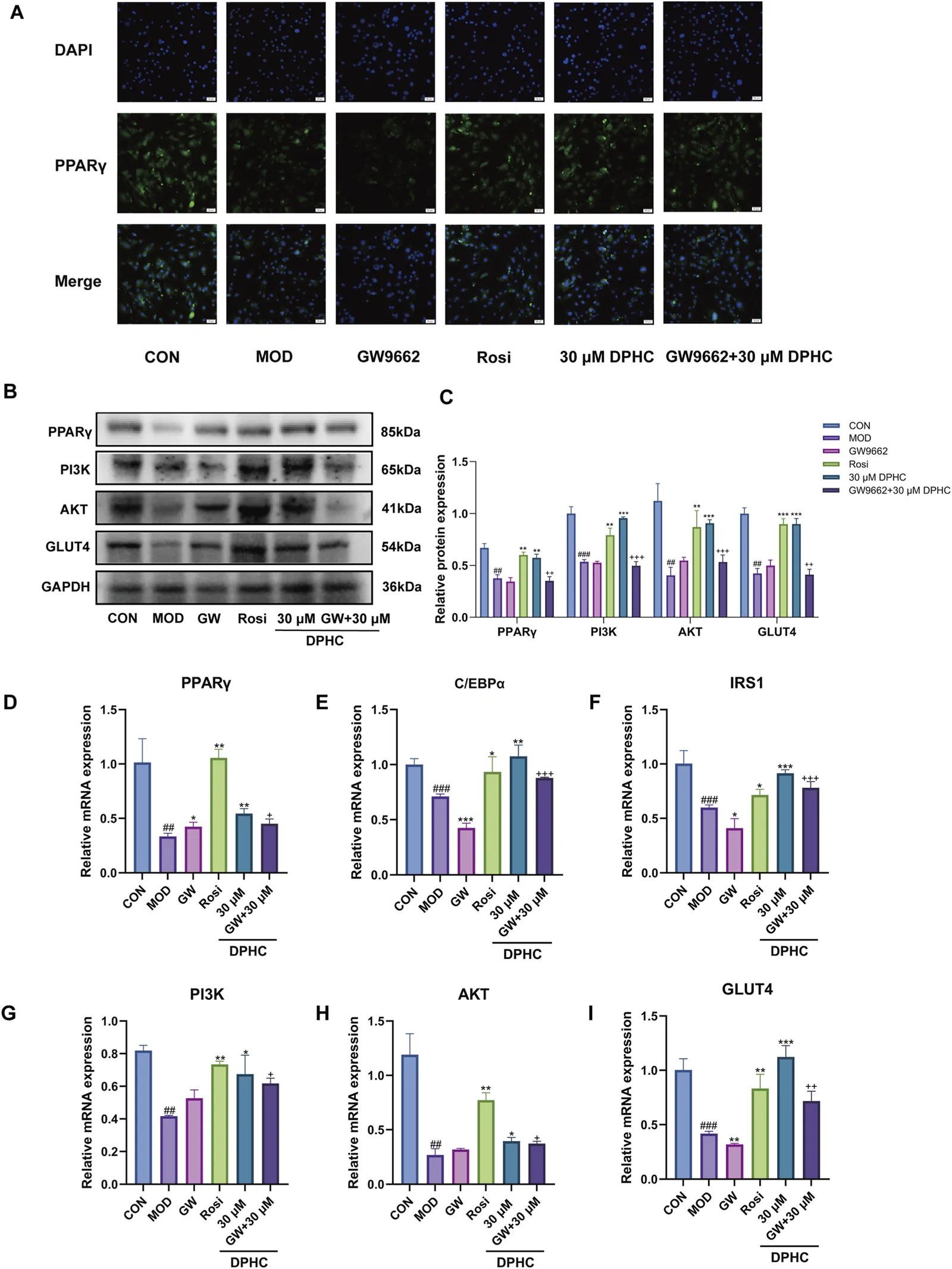
GW9662 attenuated DPHC-associated changes in PPARγ and insulin-signaling-related expression in IR-3T3-L1 adipocytes. **(A)** Immunofluorescence staining of PPARγ (scale bar = 50 μm). **(B)** Western blot analysis. PPARγ, PI3K, AKT, and GLUT4 protein expressions. **(C)** Quantitative analysis of PPARγ, PI3K, AKT, and GLUT4 protein expressions. **(D–I)** qPCR analysis. Relative mRNA expressions of PPARγ, C/EBPα, IRS1, PI3K, AKT, and GLUT4. All data are expressed as the mean ± SD (n = 3). ^###^P < 0.001, ^##^P < 0.01 vs. the CON group; ^***^P < 0.001, ^**^P < 0.01 and ^*^P < 0.05 vs. the MOD group; ^+++^P < 0.001, ^++^P < 0.01 and ^+^P < 0.05 vs. the 30 μM DPHC group.

### 3.3 *In vivo* assays

#### 3.3.1 Effect of DPHC on the lipid accumulation in adipose and liver tissues of db/db mice

Lipid accumulation levels in adipose and liver tissues of db/db mice were determined by Oil Red O staining. Compared with the db/m group, lipid droplets in adipose and liver tissues of the db/db group were significantly increased, and the lipid droplets became larger. Cell morphology in liver tissues changed due to excessive lipid, and lipid droplets in adipose tissues were significantly aggregated. As expected, treatment with the positive control Rosi markedly reduced lipid accumulation in both tissues. Similarly, different doses of DPHC significantly decreased lipid deposition in the liver and adipose tissues, showing a trend consistent with that observed in the Rosi group (Figures 8A,B). The cell morphology in liver was significantly improved, the distribution of lipid droplets was no longer dense, and the lipid droplets in adipose tissues became significantly smaller and the aggregation was significantly improved. These results suggest that DPHC effectively decreased the ectopic accumulation of lipid in a dose-dependent manner, and reduced the tissue damage and lipotoxicity of excessive lipid to insulin target organs.

**FIGURE 8:**
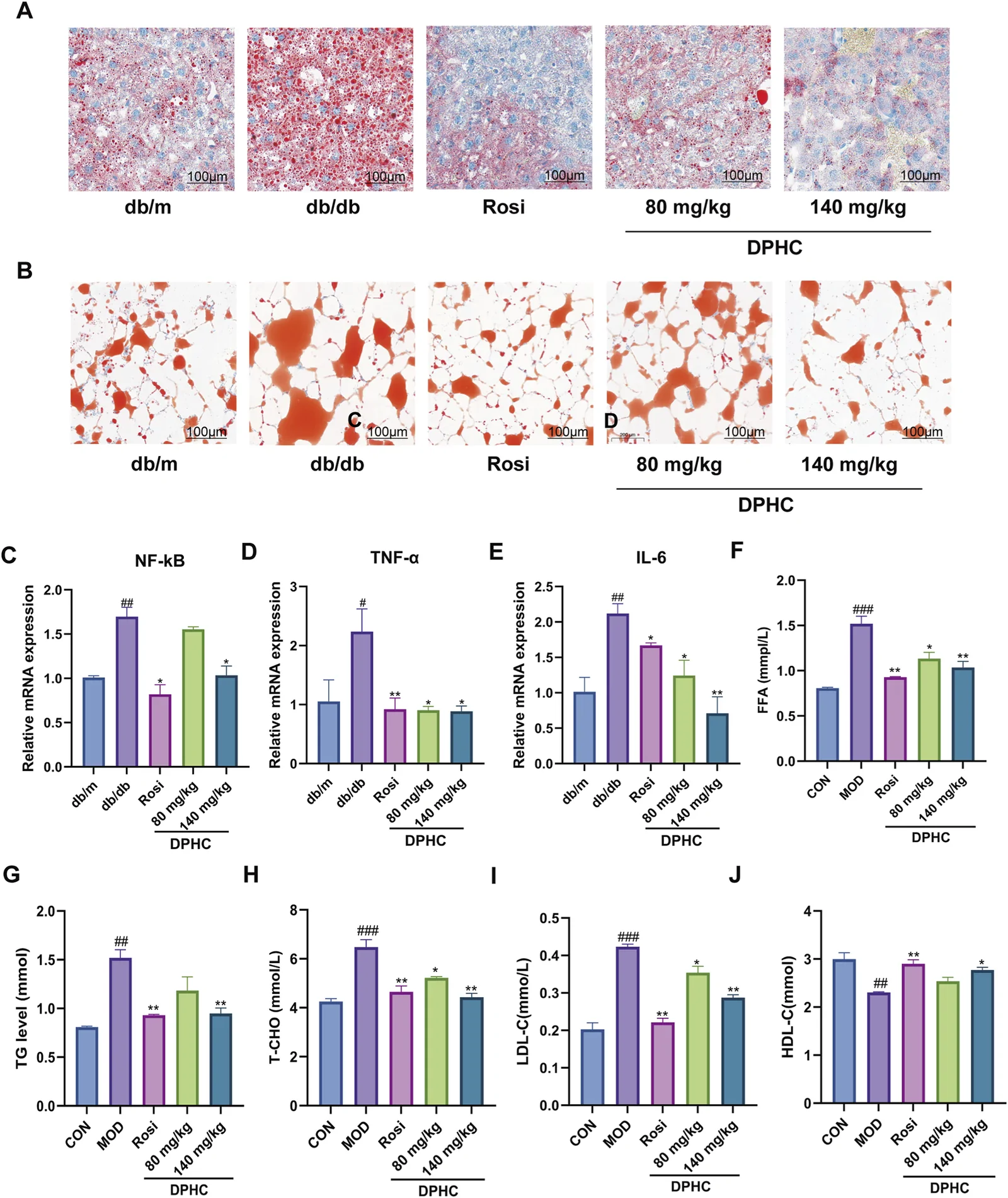
Effects of DPHC on lipid accumulation, adipokines and lipid metabolites in db/db mice. **(A)** The Oil Red O staining in liver tissues (scale bar = 100 μm). **(B)** The Oil Red O staining in adipose tissues (scale bar = 100 μm). **(C–E)** qPCR analysis. Relative mRNA expressions of adipokines including NF-κB, TNF-α, and IL-6. **(F–J)** Serum lipid profile levels, including FFA, TG, T-CHO, LDL-C and HDL-C. All data are expressed as the mean ± SD (n = 9). ^###^P < 0.001, ^##^P < 0.01 and ^#^P < 0.05 vs. the db/m group; ^**^P < 0.01 and ^*^P < 0.05 vs. the db/db group.

#### 3.3.2 Effect of DPHC on the adipokines and lipid metabolites in db/db mice

Our previous research has reported that DPHC could ameliorate glucose tolerance and IR, thereby accelerated glucose metabolism and reduced fasting blood glucose level in db/db mice (Zhang et al., 2022). However, the role of DPHC in improving IR by regulating lipid metabolism in adipose tissues of db/db mice remained unclear. Therefore, we investigated the effects of DPHC on the adipokines in adipose tissues of db/db mice. Our research indicated that the levels of TG, NF-κB, TNF-α, and IL-6 were markedly elevated in the db/db group when in comparison to the db/m group as illustrated in Figures 8C–E (P < 0.001), indicating that IR increased the secretion of adipokines and exacerbated the inflammatory response associated with T2DM. Conversely, Rosi administration significantly reduced the levels of these adipokines when the Rosi group was compared to db/db group (P < 0.05). Interestingly, DPHC also played a role in reversing the increase in adipokines in the adipose tissues of db/db mice. As such, DPHC has the potential to enhance insulin sensitivity by modulating TG level and the secretion of inflammatory factors.

In addition, we examined the effect of DPHC on lipid metabolite levels in serum of db/db mice. As shown in Figures 8F–J, compared with the db/m group, the levels of FFA, TG, T-CHO and LDL-C in the serum of the db/db group were significantly increased, while the level of HDL-C was significantly decreased, proving that IR aggravated lipid metabolism disorders in db/db mice. Lipid hypermetabolism in db/db mice under IR state leads to the increase of free lipid metabolites in blood, which increases the risk of ectopic lipid deposition and lipotoxicity. After treatment with DPHC and positive drug Rosi, it was found that the levels of lipid metabolites FFA, TG, T-CHO, and LDL-C in db/db mice were significantly decreased, and the level of HDL-C was significantly increased (P < 0.001), indicating that DPHC had the ability to significantly improve lipid metabolism disorders in db/db mice. DPHC can dose-dependently alleviate excessive lipid metabolism and effectively reduce the accumulation of lipid metabolites in the blood.

#### 3.3.3 Effect of DPHC on the PPARγ/PI3K/AKT signaling pathway in the adipose tissues of db/db mice

In this study, the effect of DPHC on PPARγ and IRS1 protein expression in adipose and liver tissues of db/db mice was examined by immunohistochemistry. The expression of PPARγ in adipose tissue and IRS1 in liver tissue was markedly reduced in the db/db group. Treatment with the positive control Rosi restored the expression of these proteins, whereas DPHC produced a similar restorative effect (Figures 9A,B), indicating activation of key factors in the PPARγ/PI3K/AKT pathway.

**FIGURE 9:**
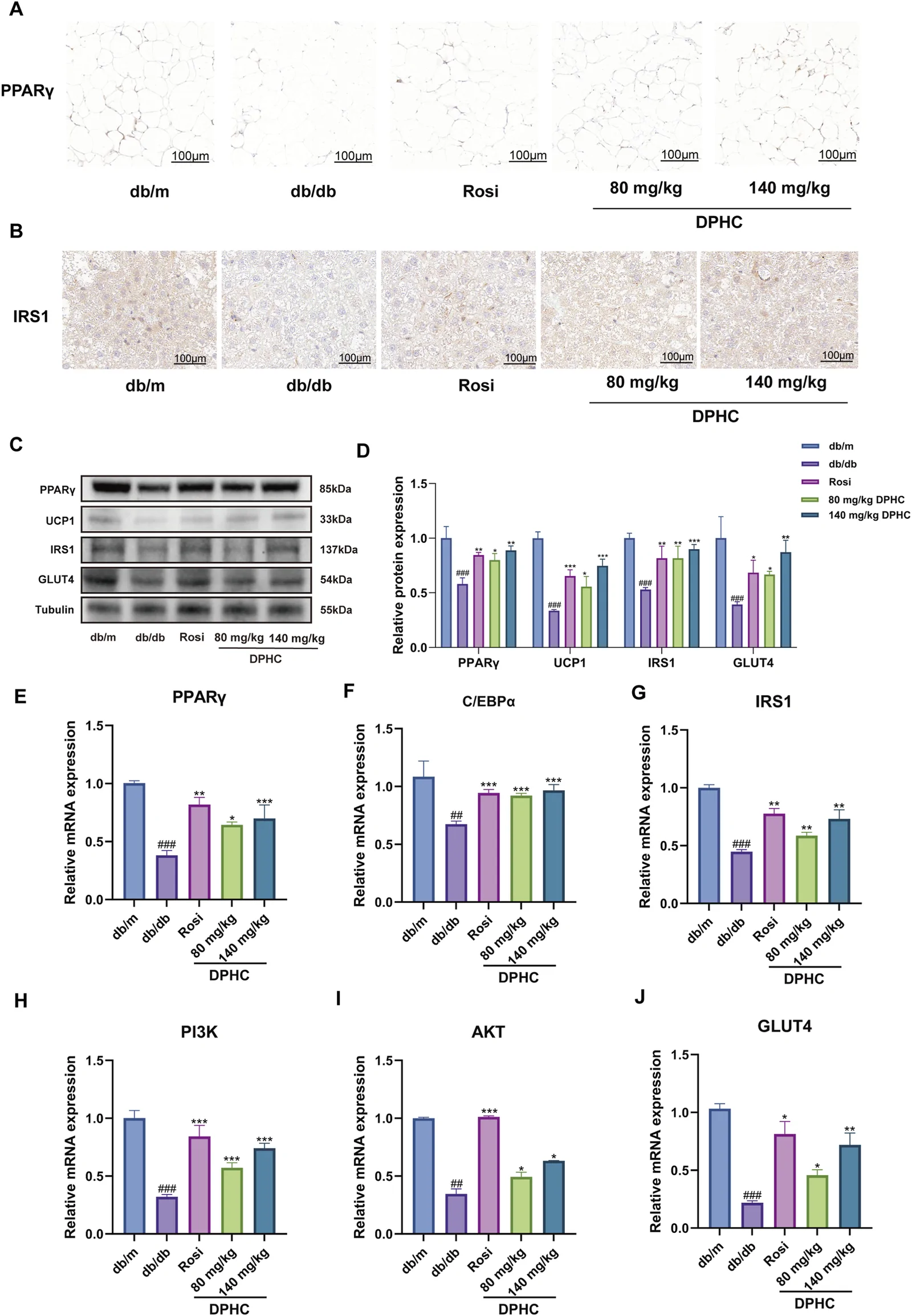
Effect of DPHC on the PPARγ/PI3K/AKT signaling pathway in the adipose tissues of db/db mice. **(A)** Immunohistochemical staining of PPARγ (IHC-epididymal adipose tissues, scale bar = 100 μm). **(B)** Immunohistochemical staining of IRS1 (IHC-liver tissues, scale bar = 100 μm). **(C)** Western blot analysis. PPARγ, UCP1, IRS1, and GLUT4 protein expressions in the adipose tissues. **(D)** Quantitative analysis of PPARγ, UCP1, IRS1, and GLUT4 protein expressions in the adipose tissues. **(E–J)** qPCR analysis. Relative mRNA expressions of PPARγ, C/EBPα, IRS1, PI3K, AKT, and GLUT4 in the adipose tissues. All data are expressed as the mean ± SD (n = 3). ^###^P < 0.001 and ^##^P < 0.01 vs. the db/m group; ^***^P < 0.001, ^**^P < 0.01 and ^*^P < 0.05 vs. the db/db group.

Besides, Western blot results showed that the expression levels of PPARγ, UCP1, IRS1 and GLUT4 proteins in the db/db group were significantly lower than those in the db/m group, whereas the positive control Rosi significantly upregulated these proteins. DPHC treatment likewise restored their expression, suggesting that DPHC may participate in biological processes such as improving glycolipid metabolism, enhancing insulin sensitivity and inhibiting inflammatory responses by up-regulating the expression of PPARγ and thereby regulating its downstream signaling molecules (Figures 9C,D).

Meanwhile, we assessed the effect of DPHC on IR and T2DM by investigating the PPARγ/PI3K/AKT signaling pathway through qPCR detection in the adipose tissues of db/db mice. Tubulin was used as an internal reference. The results of the db/m group were used to standardize the remaining groups. As depicted in Figures 9E–J, the mRNA expression levels of PPARγ, C/EBPα, IRS1, PI3K, AKT, and GLUT4 in the db/db group were observably lower than those in the db/m group (P < 0.01), suggesting that the PPARγ/PI3K/AKT signaling pathway in db/db mice with IR was severely inhibited. In contrast, after Rosi administration, the expression levels of PPARγ, C/EBPα, IRS1, PI3K, AKT, and GLUT4 were significantly increased in the Rosi group compared with the db/db group (P < 0.05). Similarly, the reduction of those mRNA levels in the db/db group was also restored and activated by oral administration of DPHC at doses of 80 and 140 mg/kg (P < 0.05), suggesting that DPHC activated the PPARγ/PI3K/AKT signaling pathway, ultimately and effectively promoting GLUT4 translocation to the cell membrane, thereby enhancing glucose metabolism rates and subsequently improving insulin sensitivity while reducing IR. Collectively, these results indicated that the alleviation of insulin resistance and lipid metabolism abnormalities by DPHC was mainly achieved through activation of the PPARγ/PI3K/AKT pathway in the adipose tissues of db/db mice.

## 4 Discussion

T2DM is a chronic metabolic disease that is mainly characterized by the persistent hyperglycemia (Wang, 2015; Galicia-Garcia et al., 2020). Current treatments for T2DM are focused on the improving effect of IR, as its occurrence can cause metabolic disorders such as hyperglycemia and hyperlipidemia (Zhu et al., 2023; Ni et al., 2024). The plant-derived natural bioactive substances have recently shown unique advantages in T2DM and IR treatment due to their minimal toxic side effects (Chen et al., 2025; Li et al., 2026; Singh et al., 2025). Our original investigation proved that DPHC from the natural plant *A. officinarum* could regulate blood glucose level and glucose tolerance in db/db mice, and simultaneously improve oxidative stress to reduce tissue damage via activating Nrf2/ARE signaling pathway (Zhang X. et al., 2022). However, IR is also closely related to lipid metabolism disorders, and whether DPHC has an effect on IR associated with lipid metabolism remains to be clarified.

IR begins in the adipose tissue, and abnormal adipose tissue differentiation, adipocyte hypertrophy, and chronic inflammation are the main causes of IR and T2DM (Li et al., 2022; Wijetunge et al., 2019). Therefore, alleviating adipose tissue accumulation and reducing inflammatory response are the key means of improving IR. In our study, DPHC treatment resulted in significantly smaller and fewer lipid droplets in IR-3T3-L1 adipocytes and adipose tissues of db/db mice, along with a reduction in the TG content, suggesting improved lipid accumulation. In addition, white adipose tissue plays an important role in the development of inflammatory response as an important organ for the production of inflammatory cytokines. Inflammatory response and abnormal lipid metabolism are the main features of metabolic diseases. Increased pro-inflammatory cytokines and the activation of inflammatory processes are the key factors involved in the pathogenesis of IR and T2DM (Nakae et al., 2008; Kawasaki et al., 2012). Adiponectin, an adipokine that is abundantly expressed in adipose tissues, has been found to increase insulin sensitivity as well as exert anti-inflammatory effects (Patappu et al., 2025). In addition, TNF-α has been closely associated with inflammation and is involved in a variety of inflammatory and immune responses (Holbrook et al., 2019). When inflammatory factors (such as TNF-α and IL-6) stimulate cells, the NF-kB P65 expression increases, which increases the inflammatory response (Sun, 2017). Our results showed that DPHC increased the secretion of adiponectin and significantly inhibited the expressions of NF-kB, TNF-α, and IL-6 in IR-3T3-L1 cells and adipose tissues of db/db mice, thereby attenuating the inflammatory response to relieve IR.

The primary etiology of IR is impaired insulin signaling, and IRS1 plays an extremely important role in the insulin-signaling pathway (Cantley, 2002; He et al., 2025; Drobiova et al., 2025). PPARγ is primarily responsible for the regulation of insulin sensitivity and adipocyte differentiation (Liu et al., 2020; Iwaki et al., 2003; Tomaru et al., 2009), and it activates the key downstream lipid metabolism transcription factor C/EBPα and reduces TG cycling by selectively promoting adipogenesis (Way et al., 2001). The activation of PPARγ improves preadipocyte differentiation and insulin sensitivity, leading to an increase in glucose uptake by adipocytes. Therefore, the enhancement of the PPARγ/PI3K/AKT signaling pathway is considered one of the effective methods for the treatment of T2DM combined with IR. Our exploratory network analysis revealed that PPARG, AKT1, and PIK3CA were the core targets of DPHC in T2DM and IR. Moreover, DPHC could promote the PPARγ, C/EBPα, IRS1, PI3K, and AKT expressions in IR-3T3-L1 cells and adipose tissues of db/db mice, in turn, which increased glucose uptake and GLUT4 translocation. Interestingly, these effects were reversed in the presence of the PPARγ antagonist GW9662, while treatment with the agonist Rosi increased the expressions of these key regulatory factors, thereby accelerating glucose utilization, enhancing insulin sensitivity and inhibiting lipid metabolism disorders. These results together demonstrated that DPHC ameliorated IR and T2DM with a specific emphasis on the PPARγ/PI3K/AKT signaling pathway.

The present findings may complement our previous report that DPHC activated the Nrf2/ARE pathway and reduced oxidative stress in liver and HepG2 models (Zhang X. et al., 2022). Oxidative stress and inflammatory signalling can impair insulin action through JNK-dependent inhibitory phosphorylation of IRS1, whereas PPARγ activation can increase adiponectin and suppress pro-inflammatory mediators. Thus, Nrf2/ARE-mediated redox control and PPARγ-mediated adipocyte regulation may converge on IRS1/PI3K/AKT/GLUT4 signalling. Consistent with possible crosstalk, Nrf2 deficiency has been reported to reduce PPARγ and C/EBPα expression and impair adipocyte differentiation (Pi et al., 2010; Kim et al., 2018). Nevertheless, the potential functional interplay between Nrf2/ARE and PPARγ signaling was not directly examined in the present study. Whether these pathways exert additive or synergistic effects on the metabolic benefits of DPHC therefore remains to be elucidated.

The exploratory network and docking analyses were used to formulate, not verify, a PPARγ-centered hypothesis. Database-derived hubs are vulnerable to study-density and tool-correlation biases (Diao et al., 2026). Consistently, the docking calculation assigned DPHC and a close analogue the same best score, and the 100-ns trajectory showed only that the selected starting pose remained geometrically stable in one simulation. The strongest mechanistic evidence in this study is therefore functional: GW9662 blunted several DPHC-associated phenotypes and expression changes. Direct engagement of PPARγ remains untested and should be assessed using a target-specific cellular engagement assay and, preferably, a purified-protein affinity method.

Several limitations warrant cautious interpretation of these findings. Direct PPARγ target engagement and binding affinity were not measured; therefore, the docking and molecular-dynamics analyses provide only a structural hypothesis rather than evidence of direct binding or agonistic activity. In addition, Although DPHC was administered at 80 and 140 mg/kg/day, systemic exposure, pharmacokinetics, dedicated toxicology, and hepatic and renal safety parameters were not evaluated. Thus, the present findings concern an isolated experimental metabolite and should not be extrapolated to clinical efficacy, clinical dosing, or systemic safety. Future studies should include direct target-engagement assays, pathway-specific functional assays, dose-ranging and toxicokinetic studies, hepatic and renal biochemical measurements and histopathological evaluation before clinical translation.

Overall, DPHC was associated with improved metabolic phenotypes in dexamethasone-induced insulin-resistant 3T3-L1 adipocytes and db/db mice, including increased glucose utilization, reduced lipid accumulation, and lower inflammatory-marker expression. Exploratory network analysis nominated PPARG, AKT1, and PIK3CA as candidate proteins, whereas molecular docking and molecular-dynamics simulations generated a candidate structural pose of DPHC within the PPARγ ligand-binding pocket; these computational findings did not demonstrate direct binding or affinity. DPHC increased the mRNA expression of PPARγ, C/EBPα, IRS1, PI3K, AKT, and GLUT4 and increased the protein expression of selected PPARγ- and insulin-signaling-associated proteins. GW9662 attenuated several cellular effects of DPHC, consistent with the involvement of a PPARγ-dependent component. Accordingly, the present findings support a working model, summarized in Figure 10, in which DPHC may alleviate adipose insulin resistance and associated lipid metabolic abnormalities partly through PPARγ-associated PI3K/AKT/GLUT4 signaling. This model requires further confirmation through direct target-engagement and pathway-specific functional studies.

**FIGURE 10:**
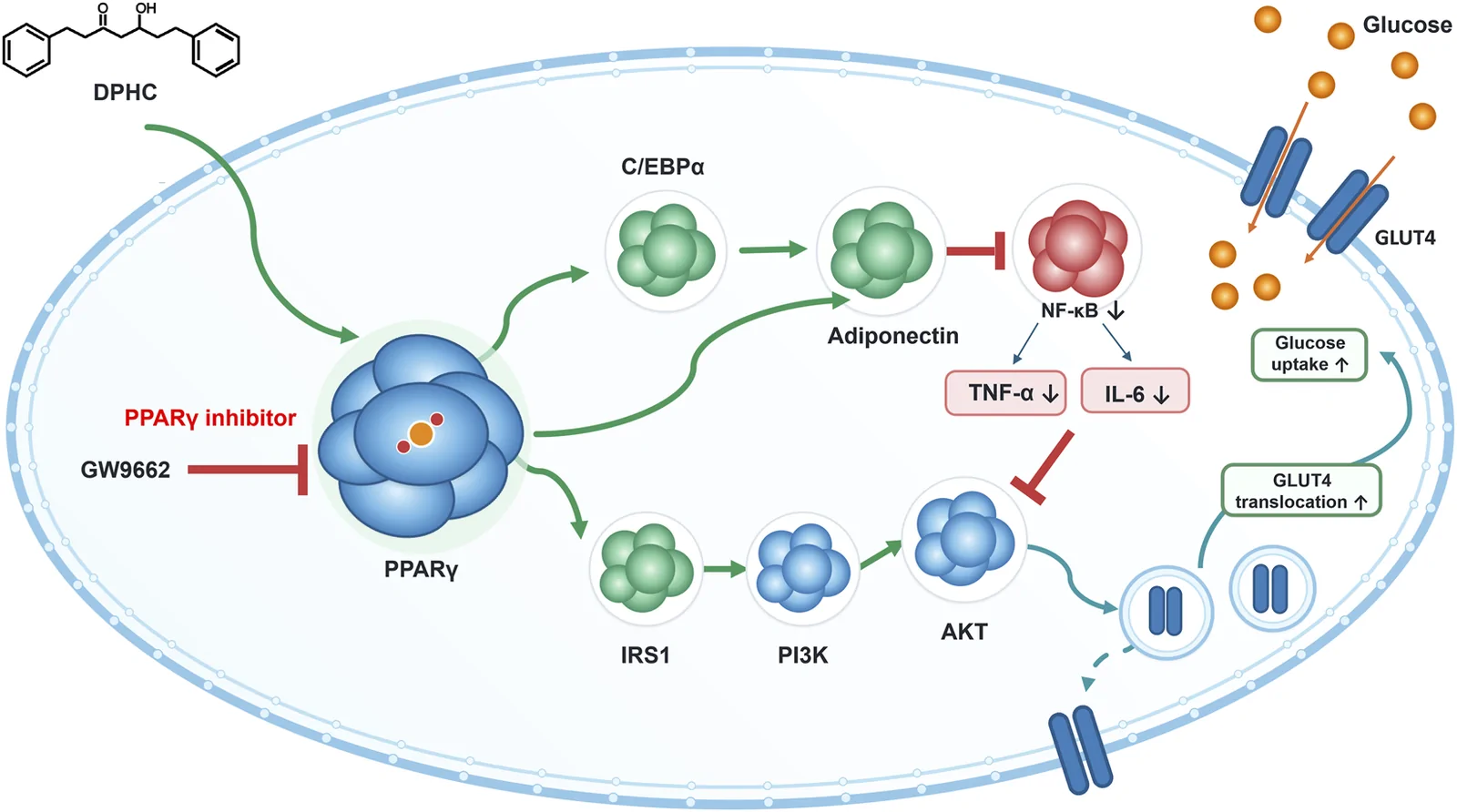
Schematic illustration of the proposed mechanism by which DPHC, derived from *Alpinia officinarum* Hance, alleviates T2DM by improving adipose IR via activation of the PPARγ/PI3K/AKT pathway.

## 5 Conclusion

This study revealed that DPHC from *A. officinarum* alleviated T2DM by improving adipose IR through the upregulation of PPARγ and the activation of insulin signaling pathway. Pharmacological inhibition of PPARγ attenuated the beneficial effects of DPHC, supporting the involvement of PPARγ-associated signaling in the improvement of lipid metabolism disorders and IR. These findings demonstrate a novel mechanism linking DPHC with T2DM pathophysiology and suggest that modulation of the PPARγ/PI3K/AKT signaling pathway may offer a promising therapeutic strategy for metabolism-related IR.

## Data Availability

The original contributions presented in the study are included in the article/Supplementary Material, further inquiries can be directed to the corresponding authors.

## References

[B1] Abraham M. J. Murtola T. Schulz R. Páll S. Smith J. C. Hess B. (2015). GROMACS: high performance molecular simulations through multi-level parallelism from laptops to supercomputers. SoftwareX 1–2, 19–25. 10.1016/j.softx.2015.06.001

[B2] Accili D. Deng Z. Liu Q. (2025). Insulin resistance in type 2 diabetes mellitus. Nat. Rev. Endocrinol. 21 (7), 413–426. 10.1038/s41387-026-00426-w 40247011

[B3] Cantley L. C. (2002). The phosphoinositide 3-Kinase pathway. Science 296 (5573), 1655–1657. 10.1126/science.296.5573.1655 12040186

[B5] Chao M. Zou D. Zhang Y. Chen Y. Wang M. Wu H. (2009). Improving insulin resistance with traditional Chinese medicine in type 2 diabetic patients. Endocrine 36 (2), 268–274. 10.1007/s12020-009-9229-5 19728183

[B6] Chen L. He T. Han Y. Sheng J. Z. Jin S. Jin M. W. (2011). Pentamethylquercetin improves adiponectin expression in differentiated 3T3-L1 cells via a mechanism that implicates PPARγ together with TNF-α and IL-6. Molecules 16 (7), 5754–5768. 10.3390/molecules16075754 21734632 PMC6264445

[B7] Chen J. Zhang M. Yang Y. Peng H. Zhang J. Shou Y. (2025). A novel glucomannan from Dendrobium officinale inhibits obesity and improves insulin sensitivity through regulating adipogenesis and lipid metabolism. J. Agric. Food Chem. 73 (43), 27751–27770. 10.1021/acs.jafc.5c06789 41104439

[B8] Cho H. Lai C. C. Bonnavion R. Alnouri M. W. Wang S. Roquid K. A. (2025). Endothelial insulin resistance induced by adrenomedullin mediates obesity-associated diabetes. Science 387 (6734), 674–682. 10.1126/Science.adr4731 39913566

[B9] Diao X. Zhang H. Wang S. Wang Z. Zhang Q. (2026). Rethinking network analysis in ethnopharmacology: a multi-omics and AI roadmap to overcome conceptual and methodological biases. Front. Pharmacol. 17, 1748478. 10.3389/fphar.2026.1748478 41800093 PMC12962898

[B10] Ding K. Zhang Z. Han Z. Shi L. Li X. Liu Y. (2025). Liver ALKBH5 regulates glucose and lipid homeostasis independently through GCGR and mTORC1 signaling. Science 387 (6737), eadp4120. 10.1126/Science.adp4120 40014709

[B11] Drobiova H. Alhamar G. Ahmad R. Al-Mulla F. Al Madhoun A. (2025). GLUT4 trafficking and storage vesicles: molecular architecture, regulatory networks, and their disruption in insulin resistance. Int. J. Mol. Sci. 26 (15), 7568. 10.3390/ijms26157568 40806697 PMC12347429

[B12] Galicia-Garcia U. Benito-Vicente A. Jebari S. Larrea-Sebal A. Siddiqi H. Uribe K. B. (2020). Pathophysiology of type 2 diabetes mellitus. Int. J. Mol. Sci. 21 (17), 6275. 10.3390/ijms21176275 32872570 PMC7503727

[B15] He W. Barak Y. Hevener A. Olson P. Liao D. Le J. (2003). Adipose-specific peroxisome proliferator-activated receptor γ knockout causes insulin resistance in fat and liver but not in muscle. Proc. Natl. Acad. Sci. U. S. A. 100 (26), 15712–15717. 10.1073/pnas.0304384100 14660788 PMC307633

[B16] He Y. Yang X. He X. Wang G. Li C. Yuan P. (2025). Mechanisms and therapeutics of insulin signaling transduction genes in diabetic cardiomyopathy: a comprehensive updated review. Front. Endocrinol. 16, 1589695. 10.3389/fendo.2025.1589695 40747301 PMC12310498

[B17] Holbrook J. Lara-Reyna S. Jarosz-Griffiths H. McDermott M. (2019). Tumour necrosis factor signalling in health and disease. Review. 8, 10.12688/f1000research.17023.1 30755793 PMC6352924

[B18] Iwaki M. Matsuda M. Maeda N. Funahashi T. Matsuzawa Y. Makishima M. (2003). Induction of adiponectin, a fat-derived antidiabetic and antiatherogenic factor, by nuclear receptors. Diabetes 52 (7), 1655–1663. 10.2337/diabetes.52.7.1655 12829629

[B19] Jangra A. Babu B. Divakar S. Gowramma B. Rajan S. Jangra S. (2025). An in-depth review of PPARγ modulators as anti-diabetes therapeutics. Drug Metab. Rev. 57 (4), 311–337. 10.1080/03602532.2025.2475743 40406962

[B20] Kalyani R. R. Neumiller J. J. Maruthur N. M. Wexler D. J. (2025). Diagnosis and treatment of type 2 diabetes in adults: a review. Jama 334 (11), 984–1002. 10.1001/Jama.2025.5956 40549398 PMC13014273

[B21] Karampetsou N. Tzani A. Doulamis I. P. Bletsa E. Minia A. Pliaka V. (2022). Epicardial Adipocyte-derived TNF-α modulates local inflammation in patients with advanced coronary artery disease. Curr. Vasc. Pharmacol. 20 (1), 87–93. 10.2174/1570161119666211029110813 34719373

[B22] Katafuchi T. Holland W. L. Kollipara R. K. Kittler R. Mangelsdorf D. J. Kliewer S. A. (2018). PPARγ-K107 SUMOylation regulates insulin sensitivity but not adiposity in mice. Proc. Natl. Acad. Sci. U. S. A. 115 (48), 12102–12111. 10.1073/pnas.1812929115 30420515 PMC6275522

[B23] Kawasaki N. Asada R. Saito A. Kanemoto S. Imaizumi K. (2012). Obesity-induced endoplasmic reticulum stress causes chronic inflammation in adipose tissue. Sci. Rep. 2 (1), 799. 10.1038/srep00799 23150771 PMC3495279

[B24] Kim B. R. Lee G. Y. Yu H. Maeng H. J. Oh T. J. Kim K. M. (2018). Suppression of Nrf2 attenuates adipogenesis and decreases FGF21 expression through PPAR gamma in 3T3-L1 cells. Biochem. Biophys. Res. Commun. 497 (4), 1149–1153. 10.1016/j.bbrc.2017.01.107 28131830

[B25] Lee J. Kim K. A. Jeong S. Lee S. Park H. J. Kim N. J. (2009). Anti-inflammatory, anti-nociceptive, and anti-psychiatric effects by the rhizomes of *Alpinia officinarum* on complete Freund's adjuvant-induced arthritis in rats. J. Ethnopharmacol. 126 (2), 258–264. 10.1016/j.jep.2009.08.033 19715749

[B26] Li X. Wen H. Zhang Y. Liu A. Zhang X. Fu M. (2022). DPHB, a diarylheptane from *Alpinia officinarum* hance, ameliorates insulin resistance: a network pharmacology and *in vitro* study. Front. Pharmacol. 13, 956812. 10.3389/fphar.2022.956812 36120365 PMC9475175

[B27] Li Y. Kang S. Wang Q. Zeng C. Dong G. (2026). Bidens biternata (Lour.) Merr. and Sherff improves liver lipid metabolism disorders in type 2 diabetic mice by activating mitophagy. J. Ethnopharmacol. 357, 120877. 10.1016/j.jep.2024.120877 41213441

[B28] Lindorff-Larsen K. Piana S. Palmo K. Maragakis P. Klepeis J. L. Dror R. O. (2010). Improved side-chain torsion potentials for the Amber ff99SB protein force field. Proteins 78, 1950–1958. 10.1002/prot.22711 20408171 PMC2970904

[B29] Liu T. Zhu C. Duan Z. Ma P. Ma X. Fan D. (2024). Network pharmacological analysis combined with experimental verification to explore the effect of ginseng polypeptide on the improvement of diabetes symptoms in db/db mice. J. Agric. Food Chem. 72 (33), 18537–18551. 10.1021/acs.jafc.4c04949 39129180

[B56] Liu Z. Xu L. Xing M. Xu X. Wei J. Wang J. (2020). Trelagliptin succinate: DPP-4 inhibitor to improve insulin resistance in adipocytes. Biomed. Pharmacother. 125, 109952. 10.1016/j.biopha.2020.109952 32036216

[B30] Moreno-Indias I. Cardona F. Tinahones F. J. Queipo-Ortuño M. I. (2014). Impact of the gut microbiota on the development of obesity and type 2 diabetes mellitus. Front. Microbiol. 5, 190. 10.3389/fmicb.2014.00190 24808896 PMC4010744

[B31] Nakae J. Cao Y. Oki M. Orba Y. Sawa H. Kiyonari H. (2008). Forkhead transcription factor FoxO1 in adipose tissue regulates energy storage and expenditure. Diabetes 57 (3), 563–576. 10.2337/db07-1465 18162510

[B32] Ni Y. Wu X. Yao W. Zhang Y. Chen J. Ding X. (2024). Evidence of traditional Chinese medicine for treating type 2 diabetes mellitus: from molecular mechanisms to clinical efficacy. Pharm. Biol. 62 (1), 592–606. 10.1080/13880209.2024.2376311 PMC11262228 39028269

[B33] Oguntibeju O. O. (2019). Type 2 diabetes mellitus, oxidative stress and inflammation: examining the links. Int. J. Physiol. Pathophysiol. Pharmacol. 11 (3), 45–63. 31333808 PMC6628012

[B34] Paprocki J. D. Macdonald P. J. Xu Y. Cheng A. Dunn T. C. Tetin S. Y. (2025). Quantifying glucose uptake at the single cell level with confocal microscopy reveals significant variability within and across individuals. Sci. Rep. 15 (1), 2661. 10.1038/s41598-025-86813-2 PMC11751187 39837851

[B35] Patappu L. Dhas P. K. Mekkhala K. P. Ponnudhali D. Jaishankar T. Saranya T. (2025). Multifaceted mechanisms of adiponectin on cardiovascular health: a comprehensive review. Biomed. Sci. Clin. Med. 64 (4), 326–335. 10.12982/BSCM.2025.30

[B36] Peng X. Chen R. Wu Y. Huang B. Tang C. Chen J. (2015). PPARγ–PI3K/AKT–NO signal pathway is involved in cardiomyocyte hypertrophy induced by high glucose and insulin. J. Diabetes Complicat. 29 (6), 755–760. 10.1016/j.jdiacomp.2015.04.012 26045205

[B37] Pi J. Leung L. Xue P. Wang W. Hou Y. Liu D. (2010). Deficiency in the nuclear factor E2-related factor-2 transcription factor results in impaired adipogenesis and protects against diet-induced obesity. J. Biol. Chem. 285 (12), 9292–9300. 10.1074/jbc.M109.093955 20089859 PMC2838347

[B38] Ramos P. A. Lytle K. A. Delivanis D. Nielsen S. LeBrasseur N. K. Jensen M. D. (2021). Insulin-stimulated muscle glucose uptake and insulin signaling in lean and obese humans. J. Clin. Endocrinol. Metab. 106 (4), 1631–1646. 10.1210/clinem/dgaa919 33382888 PMC7993573

[B39] Shimizu M. Yamashita D. Yamaguchi T. Hirose F. Osumi T. (2006). Aspects of the regulatory mechanisms of PPAR functions: analysis of a bidirectional response element and regulation by sumoylation. Mol. Cell. Biochem. 286 (1-2), 33–42. 10.1007/s11010-005-9029-5 16534556

[B40] Singh S. S. Eligar S. M. Patil K. N. (2025). Ferulic acid from beta vulgaris subsp. vulgaris altissima group pulp targets the CaMKKβ/SIRT-1/AMPK pathway to combat hyperglycemia. J. Agric. Food Chem. 73 (36), 22347–22361. 10.1021/acs.jafc.4c07776 40884506

[B41] Sun S. C. (2017). The non-canonical NF-κB pathway in immunity and inflammation. Nat. Rev. Immunol. 17, 545–558. 10.1038/nri.2017.47 28580957 PMC5753586

[B42] Sylow L. Tokarz V. L. Richter E. A. Klip A. (2021). The many actions of insulin in skeletal muscle, the paramount tissue determining glycemia. Cell Metab. 33 (4), 758–780. 10.1016/j.cmet.2021.03.004 33826918

[B43] Tomaru T. Steger D. J. Lefterova M. I. Schupp M. Lazar M. A. (2009). Adipocyte-specific expression of murine resistin is mediated by synergism between peroxisome proliferator-activated receptor gamma and CCAAT/enhancer-binding proteins. J. Biol. Chem. 284 (10), 6116–6125. 10.1074/jbc.M807215200 19126543 PMC2649096

[B44] Trott O. Olson A. J. (2010). AutoDock Vina: improving the speed and accuracy of docking with a new scoring function, efficient optimization, and multithreading. J. Comput. Chem. 31, 455–461. 10.1002/jcc.21334 19499576 PMC3041641

[B45] Wang X. L. (2015). Practical Molecular Pharmacology. Beijing: China Union Medical University Press, 310.

[B46] Wang J. Wolf R. M. Caldwell J. W. Kollman P. A. Case D. A. (2004). Development and testing of a general Amber force field. J. Comput. Chem. 25, 1157–1174. 10.1002/jcc.20035 15116359

[B48] Wang L. W. Gao H. B. Yu Q. (2022). Clinical effects of metformin and canagliflozin combined with intensive insulin in the treatment of newly diagnosed type 2 diabetes mellitus. Chin. Foreign Med. Res. 20 (7), 30–34. 10.14033/j.cnki.cfmr.2022.07.008

[B50] Way J. M. Harrington W. W. Brown K. K. Gottschalk W. K. Sundseth S. S. Mansfield T. A. (2001). Comprehensive messenger ribonucleic acid profiling reveals that peroxisome proliferator-activated receptor gamma activation has coordinate effects on gene expression in multiple insulin-sensitive tissues. Endocrinology 142 (3), 1269–1277. 10.1210/endo.142.3.8039 11181544

[B51] Wijetunge S. Ratnayake R. M. C. J. Kotakadeniya H. M. S. R. B. Rosairo S. Albracht-Schulte K. Ramalingam L. (2019). Association between serum and adipose tissue resistin with dysglycemia in South Asian women. Nutr. Diabetes 9 (1), 5. 10.1038/s41387-019-0073-3 30778042 PMC6379415

[B53] Yuan L. Tang P. Li H. J. Hu N. Zhong X. Y. Lin M. (2019). Serum from Jiao-Tai-Wan treated rats increases glucose consumption by 3T3-L1 adipocytes through AMPK pathway signaling. Biosci. Rep. 39 (4), BSR20181286. 10.1042/bsr20181286 30886061 PMC6449522

[B54] Zhang X. G. Liu A. X. Zhang Y. X. Zhou M. Y. Li X. Y. Fu M. H. (2022). A diarylheptanoid compound from *Alpinia officinarum* hance ameliorates high glucose-induced insulin resistance by regulating PI3K/AKT-Nrf2-GSK3β signaling pathways in HepG2 cells. J. Ethnopharmacol. 295, 115397. 10.1016/j.jep.2022.115397 35605918

[B55] Zhang X. Zhang Y. Zhou M. Xie Y. Dong X. Bai F. (2022). DPHC from *Alpinia officinarum* ameliorates oxidative stress and insulin resistance via activation of Nrf2/ARE pathway in db/db mice and high glucose-treated HepG2 cells. Front. Pharmacol. 12, 792977. 10.3389/fphar.2021.792977 35111058 PMC8801804

[B57] Zhu S. L. Xu W. H. Li B. T. Tu J. N. (2023). *In vitro* study of Pueraria Mirifica modulating PPARs signaling pathway to improve insulin resistance in adipocytes. New Chin. Med. Clin. Pharmacol. 34 (3), 287–295. (In Chinese)

