# Supplementary material for: A diarylheptanoid from Alpinia officinarum ameliorates insulin resistance in 3T3-L1 adipocytes and db/db mice through modulation of PPARγ/PI3K/AKT signaling pathway
Source: Front Pharmacol. 2026 Oct 14;17:1934799. doi: 10.3389/fphar.2026.1934799 (PMC13713682; doi:10.3389/fphar.2026.1934799)
Supplement: Supplementary file 1 [file Supplementaryfile1.docx]

Supplementary Fig. S1. Descriptive analysis of the single 100-ns DPHC–PPARγ molecular-dynamics trajectory. (A) Backbone RMSD. (B) Radius of gyration. (C) Solvent-accessible surface area (SASA). (D) Protein–ligand hydrogen-bond count. (E) Cα RMSF. (F) Two-dimensional free-energy landscape based on RMSD and radius of gyration. This simulation assesses persistence of the selected pose and does not measure binding affinity.
